# β‐Hydroxybutyrate enhances chondrocyte mitophagy and reduces cartilage degeneration in osteoarthritis via the HCAR2/AMPK/PINK1/Parkin pathway

**DOI:** 10.1111/acel.14294

**Published:** 2024-08-09

**Authors:** Huangming Zhuang, Xunshan Ren, Yuelong Zhang, Huajie Li, Panghu Zhou

**Affiliations:** ^1^ Department of Orthopedics Renmin Hospital of Wuhan University Wuhan China

**Keywords:** hydroxycarboxylic acid receptor 2, mitophagy, osteoarthritis, senescence, β‐Hydroxybutyrate

## Abstract

Osteoarthritis (OA) is widely recognized as the prevailing joint disease associated with aging. The ketogenic diet (KD) has been postulated to impede the advancement of various inflammatory ailments. β‐Hydroxybutyrate (βOHB), a prominent constituent of ketone bodies, has recently been proposed to possess crucial signaling capabilities. In this study, we propose to explore the role and mechanism of βOHB in OA. Tissue staining and inflammatory factor assay were employed to evaluate the impacts of KD and βOHB on OA rats. The oxidative stress conditions in chondrocytes were induced using tert‐butyl hydroperoxide (TBHP). The mechanisms were determined using the siRNA of hydroxycarboxylic acid receptor 2 (HCAR2), the antagonist of adenosine monophosphate‐activated protein kinase (AMPK), and the inhibitor of mitophagy. The administration of KD demonstrated a reduction in pathological damage to cartilage, as well as a decrease in plasma levels of inflammatory factors. Furthermore, it resulted in an increase in the concentration of βOHB in the blood and synovial fluid. In vitro experiments showed that βOHB facilitated mitophagy and adenosine triphosphate production. Besides, βOHB mitigated chondrocyte senescence, inflammatory factors secretion, extracellular matrix degradation, and apoptosis induced by TBHP. Subsequent investigations indicated that the protective effects of βOHB were no longer observed following the knockdown of HCAR2, the antagonist of AMPK, or the inhibitor of mitophagy. Moreover, in vivo studies suggested that βOHB played a protective role by targeting the HCAR2‐AMPK‐PINK1 axis. In conclusion, βOHB enhanced chondrocyte mitophagy through the HCAR2/AMPK/PINK1/Parkin pathway, offering a potential therapeutic approach for the treatment of OA.

## Graphical abstract

The intervention with tert‐butyl hydroperoxide induced the production of reactive oxygen species in chondrocytes, which subsequently resulted in a reduction of mitochondrial membrane potential, chondrocyte senescence, degradation of the extracellular matrix, release of inflammatory factors, and apoptosis. Conversely, treatment with β‐Hydroxybutyrate ameliorated chondrocyte injury by activating hydroxycarboxylic acid receptor 2, promoting adenosine monophosphate‐activated protein kinase phosphorylation, and thereby facilitating PINK1‐mediated mitophagy.

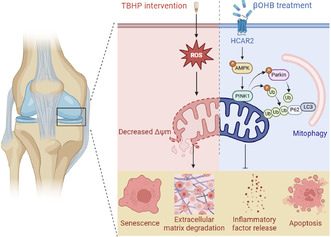

**Abbreviations**
βOHB: β‐Hydroxybutyrate
Δψm: mitochondrial membrane potential
ACLT: anterior cruciate ligament transection
AMPK: adenosine monophosphate‐activated protein kinase
ATP: adenosine triphosphate
BAX: BCL2‐associated X protein
BCL2: B‐cell lymphoma 2
CAT: catalase
CCK‐8: cell counting kit‐8
cleaved PARP‐1: cleaved polyADP‐ribose polymerase 1
Col2a1: collagen type II alpha 1
H&E: hematoxylin–eosin
HCAR2: hydroxycarboxylic acid receptor 2
IL‐6: interleukin‐6
iNOS: inducible nitric oxide synthase
KD: ketogenic diet
LC3B: microtubule‐associated protein 1 light chain 3 beta
Mdivi‐1: mitochondrial division inhibitor‐1
MMP13: matrix metalloproteinase 13
OA: osteoarthritis
OARSI: Osteoarthritis Research Society International
OCR: Oxygen consumption rate
P16: cyclin‐dependent kinase 4 inhibitor
P21: cyclin‐dependent kinase inhibitor 1A
p‐AMPK: phospho‐AMPKα Thr172
PBS: phosphate‐buffered saline
PGE2: prostaglandin E2
PINK1: PTEN induced putative kinase 1
pNA: p‐nitroaniline
p‐Parkin: phospho‐Parkin‐Ser65
p‐Ub: phospho‐Ubiquitin‐Ser65
RNS: reactive nitrogen species
ROS: reactive oxygen species
RT‐qPCR: reverse transcription–quantitative polymerase chain reaction
SASP: senescence‐associated secretory phenotype
SA‐β‐Gal: senescence‐associated β‐galactosidase
SD: standard diet
SOD1: superoxide dismutase 1
TBHP: tert‐butyl hydroperoxide
TNF‐α: tumor necrosis factor‐α
TOMM20: translocase of outer mitochondrial membrane 20

## 1 INTRODUCTION

Osteoarthritis (OA) affects approximately 25% of individuals aged 60 and above (Hunter & Bierma‐Zeinstra, 2019). The rising incidence of OA can be attributed to factors such as obesity and the aging population, leading to a significant societal and familial burden (O'Neill et al., 2018). In cases of advanced OA, arthroplasty is often necessary to restore joint function due to the limited efficacy of available treatments (Bannuru et al., 2019). The pathogenesis of OA is multifaceted, involving various mechanisms such as chondrocyte senescence, pro‐inflammatory cytokine release, oxidative stress injury, apoptosis, impaired mitochondrial energy metabolism, and dysregulated protein metabolism (Goutas et al., 2018). These intricate etiologies interact synergistically to result in common clinical manifestations ultimately. Mitophagy is vital in maintaining mitochondrial quality control and homeostasis in vivo (Sun et al., 2021). Impairment of mitophagy leads to the gradual buildup of defective mitochondria, ultimately causing chondrocyte apoptosis, extracellular matrix degradation, and cartilage degeneration (Ansari et al., 2018).

Chondrocytes, the sole resident cells in articular cartilage, maintain cartilage homeostasis through the secretion of aggrecan and collagen type II (Zhang et al., 2023). Age is a well‐established independent risk factor for OA, characterized by the accumulation of senescent chondrocytes (Chen et al., 2021). The cell cycle is irreversibly arrested during chondrocyte senescence, and the senescence‐associated secretory phenotype (SASP) emerges (Huang et al., 2022). The activation of SASP factors induces cellular inflammatory responses, oxidative stress, and cartilage matrix degradation, indicating that targeting chondrocyte senescence is crucial for therapeutic interventions in OA (Lopez‐Otin et al., 2023).

In recent times, there has been a growing focus on the impact of dietary modifications and the resulting metabolic alterations on chondrocyte dysfunction (Nasui et al., 2022; Zheng et al., 2021). Ketogenic diet (KD), which follow a high‐fat and low‐carbohydrate diet, has been found to profoundly alter human metabolomes, particularly by increasing the levels of circulating ketone bodies (Effinger et al., 2023). The KD has demonstrated immunomodulatory properties and has shown potential in alleviating symptoms associated with several chronic ailments. However, there is still a need for further investigation regarding the therapeutic role and mechanisms of KD in OA (Abboud et al., 2021). β‐Hydroxybutyrate (βOHB) is one of the most significant components of ketone bodies (Sharma & Ramanathan, 2020). The metabolic analysis of knee synovial fluid in OA patients revealed a decrease in βOHB concentration, which exhibited a negative correlation with collagen levels (Mickiewicz et al., 2015). Recent research has highlighted the role of ketone bodies as both energy carriers and signaling molecules (Newman & Verdin, 2014). Specifically, it has been demonstrated that βOHB downregulated the production of inflammatory cytokines in microglia stimulated by lipopolysaccharide (Fu et al., 2014), inhibited the inflammatory activation of bone marrow macrophages (Youm et al., 2015), and mitigated myocardial oxidative stress in septic cardiomyopathy (Ji et al., 2022). Moreover, previous clinical investigations have demonstrated the favorable safety profile of exogenous supplementation of βOHB (Stefan et al., 2021). Considering the modulatory effects of βOHB on energy metabolism, inflammatory reactions and oxidative stress, exploring its potential therapeutic applications in OA is valuable. Hydroxycarboxylic acid receptor 2 (HCAR2), also referred to as G protein‐coupled receptor 109a (GPR109a), serves as a significant endogenous receptor for βOHB (Dmitrieva‐Posocco et al., 2022; Zhao et al., 2023). Adenosine monophosphate‐activated protein kinase (AMPK), a highly conserved master regulator of cellular energy metabolism, can be activated through the supplementation of βOHB (Carretta et al., 2020; Hardie et al., 2012). Consequently, we postulated that the activation of HCAR2 and AMPK may be intricately linked to the mechanism of action of βOHB.

This study aimed to evaluate the capacity and mechanism of βOHB in mitigating oxidative stress, mitochondrial dysfunction, cellular senescence, and apoptosis induced by tert‐butyl hydroperoxide (TBHP) in vitro. Additionally, the effects of KD and βOHB were examined in an OA rat model.

## 2 METHODS

### 2.1 Ethics statement

All experiments involving animals were conducted according to the ethical policies and procedures approved by the Laboratory Animal Welfare & Ethics Committee of the Renmin Hospital of Wuhan University (Approval No: 20220103A).

### 2.2 Cell culture

Primary chondrocytes were obtained from 8‐week‐old Wistar rats. In brief, an overdose of pentobarbital intraperitoneally was administered to the rats, followed by immersion in 75% ethanol for 10 min. Subsequently, knee cartilage was aseptically isolated and fragmented into smaller pieces. After 30 min of enzymatic digestion with 0.25% trypsin, the cartilage fragments were subjected to 8 h of enzymatic digestion with 0.1% collagenase II. Chondrocytes were harvested and cultured in media containing 10% fetal bovine serum. Our study utilized only second‐generation chondrocytes to preserve chondrocyte phenotypic characteristics.

### 2.3 Cell viability assay

For measuring the cytotoxicity of βOHB (S33155, Shyuanye) and TBHP (B106035, Aladdin), a cell counting kit‐8 (CCK‐8, G4103, Servicebio) was used. Cells were cultured at a density of 5 × 10^3^/well in 96‐well plates. Different concentrations of βOHB (0.5, 1, 2, 4, and 8 mM) or TBHP (0, 5, 10, 15, 20, and 25 μM) were added. The absorbance of each concentration was measured at 24, 48, and 72 h using a microplate reader.

### 2.4 Senescence‐associated β‐galactosidase (SA‐β‐Gal) staining and Alcian blue staining

Following a 72‐h treatment of TBHP and βOHB, chondrocytes were cultured with β‐galactosidase Staining Fixative and then stained with the senescence β‐galactosidase Staining Solution (C0602, Beyotime) overnight at 37 °C. For Alcian blue staining, chondrocytes were incubated with Alcian blue reagent (BH0004, Powerful Biology) for 1 h. Subsequently, the cells were photographed under a light microscope.

### 2.5 Reverse transcription–quantitative polymerase chain reaction (RT‐qPCR)

The intervention time for TBHP and βOHB in this study was 48 h, unless otherwise specified. The extraction of total RNA was performed using TRIzol® Reagent. Complementary DNA synthesis was performed utilizing the mRNA Reverse Transcription Kit (AG11728, Accurate Biotech). The RT‐qPCR was undertaken on LightCycler® 480 Software (Roche, Swiss Confederation) using 2 × SYBR Green qPCR Mix Kits (G3320, Servicebio). The primer sequences utilized in this investigation are provided in Table 1. The relative mRNA expression was normalized to β‐actin using the 2^−ΔΔCt^ method.

**TABLE 1:** Primer sequences used in this study.

Gene name	F/R	Sequences 5′‐3′
P16	F	GAGGACCCCACCACCCTCTC
	R	ATACCGCAAATACCGCACGA
P21	F	AGCAGTTGAGCCGCGATTG
	R	ACCCAGGGCTCAGGTAGATCTTG
Aggrecan	F	AGTGACCCATCTGCTTACCCTG
	R	CTGCATCTATGTCGGAGGTAGTG
Col2a1	F	GACTGTGCCTCGGAAGAA
	R	CTGGACGTTAGCGGTGTT
IL6	F	AAATCTGCTCTGGTCTTC
	R	AGGGTTTCAGTATTGCTC
MMP13	F	GCCACCTTCTTCTTGTTGAGTTG
	R	GACTTCTTCAGGATTCCCGCA
BAX	F	TTTTTGCTACAGGGTTTC
	R	TTGTTGTCCAGTTCATCG
BCL2	F	GAGAGCGTCAACAGGGAG
	R	GCCAGGAGAAATCAAACA
CAT	F	CAGATGTGAAGCGCTTCAACAGT
	R	GGGTGGACGTCAGTGAAATTC
SOD1	F	GCAGAAGGCAAGCGGTGAAC
	R	CCAGGTCTCCAACATGCCTC
β‐Actin	F	TGCTATGTTGCCCTAGACTTCG
	R	GTTGGCATAGAGGTCTTTACGG

Abbreviations: F, Forward; R, Reverse.

### 2.6 Immunofluorescence

Chondrocytes underwent a series of procedures, including washing with phosphate‐buffered saline (PBS), fixation with 4% paraformaldehyde, and incubation with 3% bovine serum albumin for 30 min at room temperature. Subsequently, cells were incubated with primary antibodies against collagen type II alpha 1 (Col2a1, 1:200, GB11021, Servicebio), Aggrecan (1:200, GB11373, Servicebio), translocase of outer mitochondrial membrane 20 (TOMM20, 1:250, ab186734, Abcam), PTEN induced putative kinase 1 (PINK1, 1:50, A7131, Abclonal), and microtubule‐associated protein 1 light chain 3 beta (LC3B, 1:100, A19665, Abclonal) overnight at 4°C, followed by incubation with secondary antibodies for 1 h. The nuclei were stained with 4′,6‐diamidino‐2‐phenylindole, and the fluorescence intensity was observed using a fluorescence microscope. To quantify the immunostaining, ROS, and JC‐1 staining images, images of the same size were randomly selected with the same exposure time. The fluorescence intensity of the images was converted to grayscale and evaluated using the ImageJ software (version 1.8) at the same threshold.

### 2.7 Western blotting

Total protein was extracted using a lysis buffer containing phosphorylated protease inhibitors. A BCA Protein Detection Kit (W041‐1‐1, NJJCBIO) was used to determine the protein concentration. Protein separation was accomplished by sodium dodecyl sulfate‐polyacrylamide gel electrophoresis and transferred onto 0.45 μM polyvinylidene difluoride membranes. The membranes were incubated overnight at 4°C with primary antibodies targeting cyclin‐dependent kinase 4 inhibitor (P16, 1:500, PA1210, Abmart), cyclin‐dependent kinase inhibitor 1A (P21, 1:1000, PA9426, Abmart), inducible nitric oxide synthase (iNOS, 1:1000, A14031, Abclonal), IL6 (1:1000, TD6087, Abmart), matrix metalloproteinase 13 (MMP13, 1:1000, 18165‐1, Proteintech), Cleaved Caspase‐3 (1:1000, #9661, Cell Signaling Technology), B‐cell lymphoma 2 (BCL2, 1:1000, ab196495, Abcam), BCL2‐associated X Protein (BAX, 1:1000, 50599‐2, Proteintech), Catalase (CAT, 1:1000, A11777, Abclonal), superoxide dismutase 1 (SOD1, 1:5000, ab13498, Abcam), PINK1 (1:1000, A7131, Abclonal), Parkin (1:500, A0968, Abclonal), phospho‐Parkin‐Ser65 (p‐Parkin, 1:500, PA5‐114616, Thermo Fisher), P62/SQSTM1 (1:5000, ab109012, Abcam), LC3B (1:500, A19665, Abclonal), phospho‐Ubiquitin‐Ser65 (p‐Ub, 1:500, CPA5761, Cohesion), cleaved polyADP‐ribose polymerase 1 (cleaved PARP‐1, 44‐698G, Thermo Fisher), phospho‐AMPKα Thr172 (p‐AMPK, 1:1000, 2535, Cell Signaling Tech), anti‐AMPKα (1:2000, 10929‐2‐AP, Proteintech), HCAR2 (1:1000, A15611, ABclonal, China), and β‐actin (1:3000, GB11001, Servicebio). Subsequently, the strips were incubated with a secondary antibody for 1 h. The protein immunoreactivity was then detected using the Bio‐Rad scanner and semi‐quantified by ImageJ software (version 1.8).

### 2.8 Caspase 3 activity assay

Caspase 3 activity was determined using the Caspase 3 Activity Assay Kit (C1115, Beyotime). The Caspase 3 Activity Assay Kit employs the enzymatic activity of caspase 3 on the substrate acetyl‐Asp‐Glu‐Val‐Asp p‐nitroanilide to quantify caspase 3 activity by measuring the concentration of the product, p‐nitroaniline (pNA), through absorbance readings. Chondrocytes were collected with trypsin and resuspended in cell culture medium. Then cells were washed with PBS prior to lysis using a ratio of 100 μL of lysate per 2 million cells. The lysate was then incubated on ice for 15 min and subsequently centrifuged at 4°C at 16,000*g* for 10 min. The protein concentration in each sample was determined using the Bradford method to standardize the protein concentration to 3 mg/mL. Subsequently, the protein samples were combined with assay buffer and exposed to Ac‐DEVD‐pNA (2 mM) for 120 min at 37°C. The absorbance resulting from caspase 3‐mediated production of pNA was quantified at 405 nm using a microplate reader.

### 2.9 Determination of reactive oxygen species (ROS) in chondrocytes and mitochondria

The ROS assay kit (G1706, Servicebio) was used to detect intracellular ROS levels. Briefly, chondrocytes were incubated with DCFH‐DA at a ratio of 1:1000 in serum‐free medium for 30 min at 37°C. Following this incubation period, chondrocytes were washed with serum‐free medium and observed utilizing a fluorescence microscope.

The mitochondria ROS was measured by the Mitochondrial Reactive Oxygen Species Assay Kit (BB‐46091, Bestbio). The mitochondrial ROS probe is able to permeate the cell membrane and specifically target chloromethyl functional groups within the mitochondria. Once inside the mitochondria, the probe undergoes rapid oxidation by ROS, resulting in the emission of red fluorescence that allows for visualization of the mitochondria within live cells. In short, the culture medium was removed and replaced with a solution containing the mitochondrial ROS probe at 37°C, and the cells were then incubated under light protection for 30 min. Subsequently, the cell nuclei were stained with DAPI and photographed under a laser confocal microscope.

### 2.10 Flow cytometry assay

The apoptosis ratio of each respective group was determined using the Apoptosis Assay Kit (40302ES, YEASEN) with a flow cytometer (Beckman Coulter, America). Briefly, a total of 10^5^ chondrocytes were collected using ethylenediaminetetraacetic acid‐free trypsin, washed twice with pre‐cooled PBS, and then resuspended in 100 μL of 1 × Binding Buffer. The cells were subsequently incubated with 5 μL Annexin V‐FITC and 10 μL PI staining solution in the dark at room temperature for 15 min. Following incubation, 400 μL of 1 × Binding Buffer was added to the samples, which were then analyzed using a flow cytometer within 1 h.

Reactive nitrogen species (RNS) refers to the interaction of iNOS with various compounds, including ROS, resulting in the production of highly oxidizing radicals and nitro compounds such as HOONO (Davies et al., 2008). The presence of RNS in cells was determined utilizing the Reactive Nitrogen Assay Kit (BB‐462112, Bestbio). According to the experimental protocol, cells were washed with PBS and then exposed to a working solution of RNS probe staining at 37°C. After a 30‐min incubation period shielded from light, any surplus probe that was not taken up by the cells was eliminated through PBS washing, and subsequently, 200 μL of Hanks' Balanced Salt Solution buffer was added for analysis using a flow cytometer (Beckman Coulter, America).

### 2.11 JC‐1 mitochondrial membrane potential (Δψm) assay

JC‐1 (C2003S, Beyotime), a cationic carbocyanine dye, exhibits aggregation and localization towards mitochondria. In normal state, JC‐1 predominantly exists as polymers and exhibits red fluorescence. However, during early apoptosis, the decrease in Δψm leads to a shift in JC‐1 towards monomers, resulting in green fluorescence. The chondrocytes were cultured in six‐well plates until reaching a density of 100,000 cells per well. The JC‐1 staining working solution was diluted in accordance with the recommended ratio of 1 mL of JC‐1 staining buffer for every 5 μL of JC‐1 (200×), as per the instructions provided by the reagent vendor. Following the washing of cells with PBS, a mixture of 1 mL of cell culture medium and an equal volume of 1 mL of the JC‐1 staining working solution was added, thoroughly mixed, and incubated in a cell incubator at 37°C for 20 min. After the incubation period, the supernatant was removed and the cells were washed twice with JC‐1 staining buffer. Subsequently, 2 mL of cell culture solution was added. The randomly selected fields of view were observed and photographed under a fluorescence microscope.

### 2.12 Comet assay

DNA Damage Comet Assay Kit (C2041S, Beyotime) was used to assess DNA damage in cells. A 1% normal melting point agarose gel and a 0.7% low melting point agarose gel were prepared following the guidelines provided by the reagent vendor. Subsequently, the gels were cooled in a water bath and made ready for use. Chondrocytes were washed thrice with PBS, digested with trypsin, and centrifuged to gather the cells. A 30 μL aliquot of 1% normal melting point agarose gel, previously heated to 45°C, was applied to a slide, covered with a coverslip, and allowed to solidify at 4°C for 10 min. The coverslip was then gently removed. Subsequently, 10 μL of cells were thoroughly mixed with 75 μL of pre‐warmed 0.7% low melting point agarose, and 70 μL droplets were swiftly aspirated and added to the initial gel layer. The mixture was evenly spread with a pipette tip and left at 4°C for 10 min to solidify. The lysis solution was prepared by combining Lysis Buffer with DMSO in a 9:1 ratio, and the cells on the slides were lysed at 4°C for 2 h. Following the rinsing with PBS, the slides were subsequently immersed in electrophoresis buffer for a duration of 40 min at room temperature. This step was undertaken to facilitate the denaturation of DNA double strands within the cellular material under alkaline conditions, thereby enabling the fragmented DNA molecules to migrate efficiently within the electric field. The electrophoresis process was conducted at a modest voltage of 25 V within an ice bath environment, shielded from exposure to light. Following electrophoresis, the slides were immersed in a neutral buffer at 4°C, then the buffer was removed and approximately 20 μL of propidium iodide solution was added drop by drop to the slides. The slides were then stained for 10–20 min in the absence of light. Subsequently, the slides were rinsed in ultrapure water, observed and photographed under a fluorescence microscope. Approximately 25 cells were randomly chosen from each sample, and the percentage of Tail DNA content (i.e., tail DNA intensity/cell DNA intensity) was determined.

### 2.13 Oxygen consumption rate (OCR) and adenosine triphosphate (ATP) detection by seahorse

Mitochondrial bioenergetics in chondrocytes were assessed using Seahorse Bioscience Extracellular Flux Analyzers (XFe24, Agilent, USA) and Seahorse Bioscience Cell Mito Stress Test Kit (# 103015‐100, Agilent). Following preparation of background‐corrected wells on the XF24 cell culture plate, 100 μL of chondrocytes were seeded into the remaining wells at a density of 50,000 cells per well. After allowing the cells to adhere to the plate, 150 μL of growth medium was added to achieve a uniform monolayer of cells. Once the cell confluence reached 50%, interventions were carried out based on the experimental design.

One day prior to the experiment, 1 mL of XF Calibrant was added to each well in the hydrated plate to submerge the sensor cartridge in the calibrant. The probe plate device was then hydrated overnight in a 37°C CO2‐free cell incubator. On the day of the assay, cells were incubated for 1 h in a CO2‐free incubator using DMEM Medium containing 10 mM glucose, 2 mM glutamine, and 1 mM pyruvate. Then, 1.5 μM Oligomycin, 2.0 μM FCCP, and 0.5 μM rotenone/antimycin A were sequentially added to the dosing wells of the probe plate. Subsequently, the probe plate and cell plate were placed into the instrument, and the XF Cell Mito Stress Test Kit in Wave Desktop 2.6 software was utilized as a reference. After measurement, the plates were extracted, 20 μL of RIPA buffer was introduced, and the protein concentration was determined using the BCA assay. Subsequently, the OCR measurement was standardized to the overall protein content. Basal respiration, maximal respiration, spare respiration capacity, nonmitochondrial respiration, and ATP production were calculated by consulting the formula provided on the official website of the Agilent XF Assay Learning Center (https://www.agilent.com.cn/en/product/cell‐analysis/how‐to‐run‐an‐assay).

### 2.14 Transmission electron microscopy

To observe mitochondria, chondrocytes were collected through centrifugation at 800 rpm for 3 min, followed by fixation with 2.5% glutaraldehyde twice for 30 min each, and subsequent treatment with 1% osmium tetroxide for 2 h. After dehydration, infiltration, embedding, sectioning, and staining, transmission electron microscopy (Hitachi, Japan) was employed to visualize the mitochondria. In conducting a morphometric analysis of mitochondrial cristae, measurements of cristae perimeter and density were obtained utilizing the ImageJ Multimeasure plug‐in. Mitochondria were then categorized based on the abundance of cristae, ranging from low to high (Balsa et al., 2019). Subsequently, percentage damaged mitochondria and mitophagy events were quantified.

### 2.15 Knockdown by siRNA

Chondrocytes were transfected with siRNA HCAR2 or siRNA scramble using Lipofectamine 3000 transfection reagent (Invitrogen, America) and Opti‐MEM (Invitrogen, America). The transfection mixture was added dropwise to the medium containing serum and incubated at 37°C with 5% CO_2_ for 8 h. Subsequently, the transfection reagent medium was replaced with a standard cell growth medium. After confirming the transfection efficiency of siRNA HCAR2 through western blotting, subsequent experiments were conducted.

### 2.16 Rat OA model construction

A total of seventy‐five 8‐week‐old Wistar rats were included in this study. Anterior cruciate ligament transection (ACLT) was conducted in a sterile environment and sustained for 2 weeks to establish an OA model (Jin et al., 2022).

In the initial phase of animal experimentation, 20 rats were randomly allocated into 4 groups to examine the impact of SD and a KD on the progression of OA: the sham + SD group, the sham + KD group, the ACLT + SD group, and the ACLT + KD group. The nutritional composition of the SD and KD can be found in Table 2. The βOHB Colorimetric Assay Kit (E‐BC‐K785‐M, Elabscience) operates on the principle that βOHB is oxidized by βOHB dehydrogenase, concurrently reducing NAD^+^ to NADH. This reaction subsequently induces a color change in WST‐8 to an orange‐yellow hue through the action of hydrogen‐transferring substances. The βOHB concentration can be quantified by measuring the absorbance at 450 nm, which is directly proportional to the βOHB content. In brief, samples were collected and subjected to centrifugation in a 50 kDa ultrafiltration tube at 10,000 × g for 15 min. Subsequently, 10 μL of each sample, along with varying concentrations of standards, were added to the respective wells of the enzyme assay plate. Fifty microliter of Enzyme Reagent was added to each well, followed by incubation at 37°C for 10 min Subsequently, 160 μL of chromogenic agent was added, and the mixture was incubated at 37°C for an additional 30 min. The optical density value of each well was then measured at 450 nm using a microplate reader. After 8 weeks, the rats were euthanized through cervical dislocation while under anesthesia. The concentration of tumor necrosis factor‐α (TNF‐α, ER1393, FineTest), interleukin‐6 (IL‐6, E‐EL‐R0015c, Elabscience), and prostaglandin E2 (PGE2, ER1800, FineTest) were determined using enzyme‐linked immuno sorbent assay kits, following the guidelines provided by the manufacturers. The concentration of nitric oxide was quantified using the Griess reagent (Beyotime, S0024). After 3 days of fixation with a 4% paraformaldehyde solution, the articular cartilage underwent decalcification through immersion in a 10% ethylenediaminetetraacetic acid solution and subsequent incubation for 30 days. The decalcifying solution was replaced every 3 days. Subsequently, the samples were subjected to dehydration in ethanol and embedding in paraffin wax.

**TABLE 2:** Basic nutrient content of the standard diet and ketogenic diet (per 100 g).

Component/item	Standard diet	Ketogenic diet
Energy (kJ)	1338.0	2804.0
Protein (g)	14.6	18.2
Fat (g)	4.0	65.2
Carbohydrates (g)	55.6	2.8
Dietary fibers (g)	4.6	7.4
Calcium (mg)	720.0	500.0
Phosphorus (mg)	600.0	300.0
Vitamin D (μg)	2.6	2.6

In the subsequent round of animal experiments, an additional 25 rats were randomly assigned to 5 groups: sham group, ACLT group, ACLT + 50 mg/kg βOHB group, ACLT + 100 mg/kg βOHB group, and ACLT + 200 mg/kg βOHB group. A study on atherosclerosis found that βOHB significantly reduced serum levels of inflammatory factors, mainly when administered at a dosage of 100 mg/kg/day through intragastric administration (Zhang et al., 2021). Therefore, in the ACLT + βOHB group, rats were injected with 50, 100, or 200 mg/kg of βOHB daily through the abdominal cavity after establishing the OA model. In total, 100 mg/kg of normal saline was given to both the sham and the ACLT groups through intraperitoneal injection. After 8 weeks of treatment, the rats were euthanized, and histological processing and analysis were conducted.

To further investigate, we attempted to block βOHB signaling using mepenzolate bromide (HCAR2 inhibitor), Compound C (AMPK antagonist), and mitochondrial division inhibitor‐1 (Mdivi‐1, mitochondrial division inhibitor). In the third round of animal experiments, 30 rats were randomized into 6 groups: sham group, ACLT group, ACLT + 200 mg/kg βOHB group, ACLT + 200 mg/kg βOHB + 10 mg/kg mepenzolate bromide group, ACLT + 200 mg/kg βOHB + 10 mg/kg Compound C group, and ACLT + 200 mg/kg βOHB + 2.5 mg/kg Mdivi‐1 group. In a manner consistent with the protocol employed in the second round of the animal experiments, a daily administration of 200 mg/kg of βOHB was administered to the βOHB‐supplemented group, while the group not receiving βOHB supplementation was injected with 200 mg/kg of normal saline. Administering 10 mg/kg of mepenzolate bromide via intraperitoneal injection reversed the protective effects of βOHB in an acute kidney injury model (Tran et al., 2016). Additionally, tail vein injection of 10 mg/kg Compound C inhibited the activity of AMPK (Yu et al., 2008). Mdivi‐1 is a selective inhibitor of mitochondrial dynamin‐related protein 1, which is widely used to hinder mitophagy (Liu et al., 2023). It has been demonstrated that 2.5 mg/kg Mdivi‐1 could effectively impede mitophagy without notable impact on cellular function (Hinge et al., 2020). Consequently, 10 mg/kg mepenzolate bromide (HY‐17585, MedChemExpress), 10 mg/kg Compound C (HY‐13418, MedChemExpress) or 2.5 mg/kg Mdivi‐1 (HY‐15886, MedChemExpress) was administered via intra‐articular administration an hour prior to βOHB treatment. Following an eight‐week treatment period, the rats were euthanized, and subsequent histological processing and analysis were carried out.

### 2.17 Histological assessment

Hematoxylin–eosin (H & E) and safranin O‐fast green were used to stain paraffin sections. The assessment of pathological changes was conducted by two observers using the Osteoarthritis Research Society International (OARSI) through blinded graded observations (Pritzker et al., 2006). The H & E staining protocol includes deparaffinization of paraffin‐embedded sections in xylene for a duration of 10 min, followed by hydration in a series of ethanol solutions. Subsequent steps involve incubation of the sections in hematoxylin staining solution for 5 min, followed by eosin staining for 1 min. Safranin O‐fast green staining is achieved by immersing the sections in Fast Green FCF (0.02%) for 5 min and Safranin O (0.1%) for 1 min (Liu et al., 2022). The grading system employed included Grade 0 to indicate an intact surface and cartilage, Grade 1 for an intact surface only, Grade 2 for surface discontinuity, Grade 3 for vertical fissures, Grade 4 for erosion, Grade 5 for denudation, and Grade 6 for deformation. In the context of immunohistochemistry experiments, antigen retrieval is carried out after dewaxing and rehydration by subjecting the sections to 10 mmol/L citrate buffer at 100°C for a duration of 30 min. The endogenous peroxidase activity and nonspecific antigen in the samples were blocked using a 3% hydrogen peroxide solution and serum (Du et al., 2020). Paraffin sections were subjected to incubation with primary antibodies targeting IL6 (1:50, TD6087, Abmart), Col2a1 (1:100, GB11021, Servicebio), MMP13 (1:100, 18165‐1, Proteintech), P16 (1:200, ab54210, Abcam), P21 (1:50, PA9426, Abmart), PINK1 (1:50, TD7742, Abmart), p‐Parkin (1:200, PA5‐114616, Thermo Fisher); Parkin (1:50, A0968, Abclonal), P62 (1:50, TA5384, Abmart), HCAR2 (1:50, A15611, Abclonal), BAX(1:1000, 50599‐2, Proteintech), and p‐AMPK (1:100, 2535, Cell Signaling Tech). Subsequently, the sections were treated with goat anti‐rabbit secondary antibody (1:100, G‐21234, Thermo Fisher Scientific), developed using 3,3‐diaminobenzidine solution, and counterstained with hematoxylin. Representative fields of view were captured with a light microscope. The immunohistochemistry images were quantified and analyzed using the ImageJ software (version 1.8).

### 2.18 Statistical analysis

The data were analyzed using SPSS 22.0 software and plotted using GraphPad Prism 8.0.2 software. The results were presented as mean ± standard deviation. For continuous variables, an unpaired Student's t‐test was used to compare two groups, while a one‐way ANOVA was used to compare multiple groups. For datasets with two parameters, two‐way ANOVA was applied. The nonparametric data (OARSI scores) were analyzed using the Kruskal–Wallis *H* test. Statistical significance was defined as *p* < 0.05.

## 3 RESULTS

### 3.1 KD attenuated OA progression in a rat model

**FIGURE 1:**
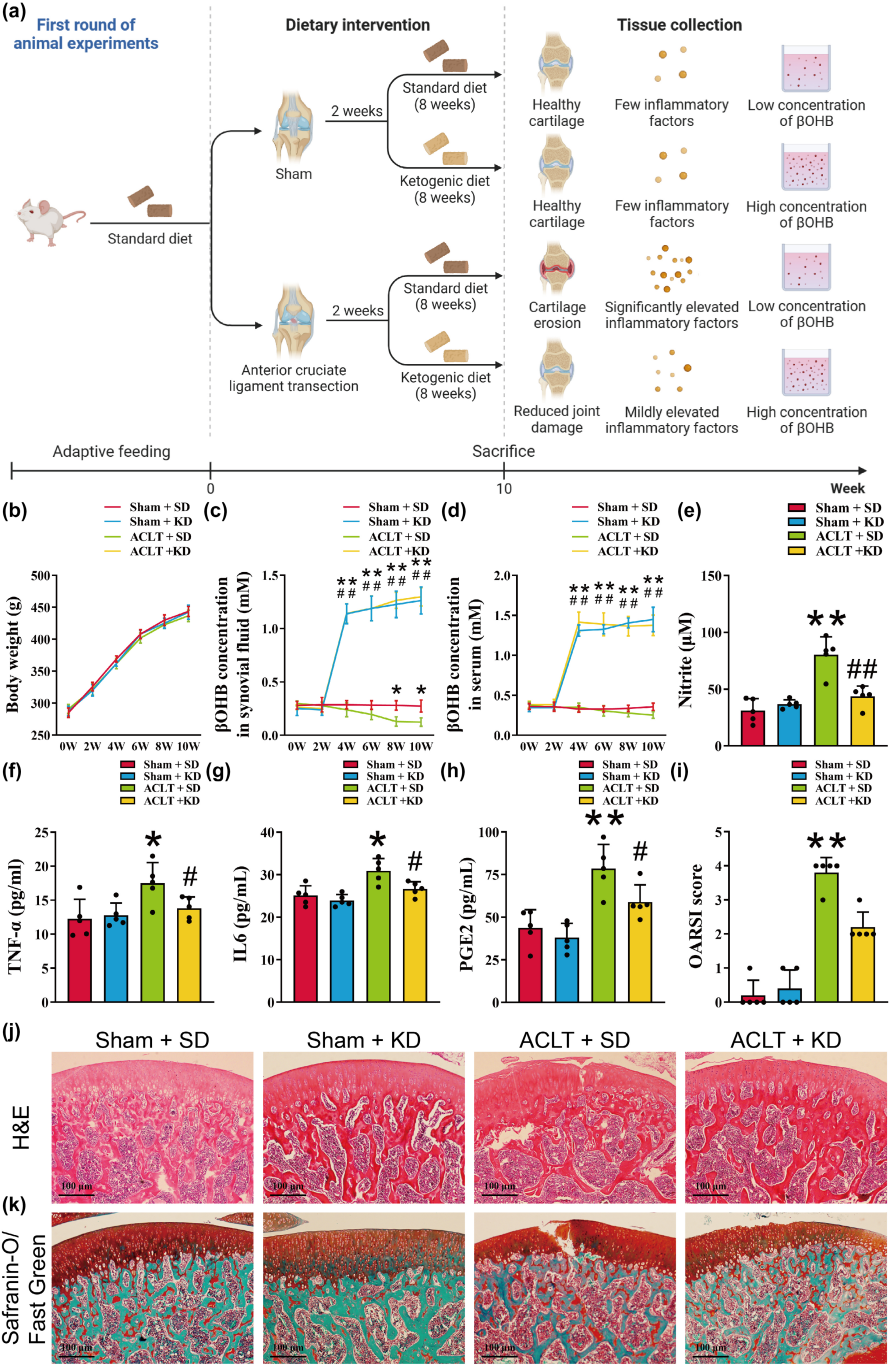
Ketogenic diet (KD) attenuated osteoarthritis (OA) progression in a rat model. (a) The schematic diagram of animal experiments. (b) The body weight of the rats was measured at various time points. (c) The βOHB levels in the synovial fluid were measured at different time points. (d) The serum βOHB levels were assessed at different time points. (e) The concentration of nitric oxide was quantified using Griess reagent. (f–h) The concentrations of interleukin‐6 (IL‐6), tumor necrosis factor‐α (TNF‐α), and prostaglandin E2 (PGE2) in the rat serum were determined through enzyme‐linked immuno sorbent assay. (i) The OARSI scores of knee cartilage were recorded. (J) Representative images of hematoxylin–eosin (H & E) staining were captured, with a scale bar of 100 μm. (k) Representative images of safranin O‐fast green staining were obtained, with a scale bar of 100 μm. *n* = 5. Data in (b–d) were analyzed by the two‐way ANOVA, data in (e–h) were analyzed by the one‐way ANOVA, and data in (i) were analyzed by the Kruskal–Wallis *H* test. *p < 0.05, **p < 0.01 versus the sham + SD group; ^#^ *p* < 0.05, ^##^ *p* < 0.01 versus the ACLT + SD group.

An OA model was built by ACLT to investigate the effects of KD on OA disease progression (Figure 1a). Neither KD nor ACLT exhibited a significant effect on the body weight of rats at any time point (Figure 1b). In the knee synovial fluid of rats subjected to the SD diet, a statistically significant decrease in βOHB concentration was observed compared to the sham‐operated group at weeks 6–8 post‐ACLT modeling (Figure 1c, *p* < 0.05). KD treatment resulted in a significant increase in βOHB concentration in both serum and synovial fluid (Figure 1c, d, *p* < 0.05). Furthermore, ACLT led to a significant increase in nitric oxide, TNF‐α, IL6, and PGE2 levels in the serum, whereas KD reversed these alterations (Figure 1e–h, *p* < 0.05). H & E staining and safranin O‐fast green staining revealed erosion and even cracking of the articular cartilage surfaces, loss of proteoglycan staining, and elevated OARSI scores in the ACLT + SD group (Figure 1i–k). However, the administration of KD resulted in the reversal of these pathological changes (Figure 1j, k). These findings indicate that KD has the potential to slow down OA progression in rats, and this effect may be attributed to the elevation of βOHB concentration in synovial fluid.

### 3.2 Effect of βOHB on chondrocyte viability

As a derivative of hydrogen peroxide, TBHP is extensively used in the modeling of osteoarthritic chondrocytes and has been shown to significantly induce oxidative stress, senescence, apoptosis, and extracellular matrix degradation in chondrocytes (He et al., 2024; Lu et al., 2021; Lv et al., 2022). To determine the optimal intervention concentration, the impact of βOHB or TBHP on chondrocyte viability was evaluated using the CCK‐8 assay at various concentrations and time points. It was observed that βOHB exhibited no significant cytotoxicity within the concentration range of 0.5–8 mM, whereas TBHP significantly decreased chondrocyte viability at concentrations exceeding 5 μM (Figure S1a, b, *p* <0.05). Furthermore, the ratio of SA‐β‐Gal positive cells increased following TBHP intervention (Figure S1c, d, *p* < 0.01), and the mRNA expression levels of P16 and P21 exhibited a significant increase in a concentration‐dependent manner (Figure S1e, *p* < 0.05). OA is a chronic degenerative disease characterized by persistent oxidative stress. To replicate this oxidative stress in vitro, we treated chondrocytes with TBHP at a relatively mild concentration of 15 μM. CCK‐8 assay results demonstrated that βOHB effectively mitigated the cytotoxic effects of TBHP, particularly within the concentration range of 1–4 mM (Figure S1f, *p* < 0.05). Given the observed HCAR2 agonist activity of βOHB at a potency of 0.9 mM (Mao et al., 2023), we selected 1 or 4 mM of βOHB and 15 μM of TBHP for subsequent chondrocytes interventions.

### 3.3 βOHB attenuates TBHP‐induced SASP in chondrocytes

We next investigated the effects of βOHB on TBHP‐induced chondrocyte senescence. SA‐β‐Gal staining revealed that βOHB effectively decreased the incidence of senescent chondrocytes in TBHP‐induced cells (Figure S2a, b, *p* < 0.01). Alcian blue staining was utilized to assess the distribution and content of glycosaminoglycans produced by cells, with a positive correlation observed between the depth of staining and the secretion of Aggrecan by chondrocytes (Yu et al., 2021). The treatment with TBHP significantly suppressed Aggrecan secretion, while the treatment with βOHB partially relieved the adverse effects of TBHP (Figure S2c, d, *p* < 0.05). Immunofluorescence staining demonstrated a decreased expression of anabolic biomarkers, including Aggrecan and Col2a1 expression, in the TBHP group compared to the control group (Figure S2e–g, *p* < 0.01). Surprisingly, the fluorescence intensity of Aggrecan and Col2a1 was restored by the treatment with βOHB (Figure S2e–g, *p* < 0.01). Furthermore, RT‐qPCR analysis revealed that TBHP intervention led to an upregulation of P16, P21, IL6, and MMP13 expression, while Aggrecan and Col2a1 expression were down‐regulated (Figure S2h–j, *p* < 0.05). The western blotting results demonstrated a significant upregulation in the protein expression of iNOS, P16, P21, IL6, and MMP13 in the TBHP group, indicating that the intervention of 15 μM TBHP induced the emergence of chondrocyte SASP phenotype (Figure S2k, l, *p* < 0.05). Besides, RT‐qPCR and western blotting results indicated that the intervention of βOHB partially mitigated the impact of TBHP on SASP‐related molecules (P16, P21, IL6, MMP13, iNOS, Aggrecan, and Col2a1) (Figure S2h–l, *p* < 0.05). The above findings provide evidence suggesting that βOHB exerts a beneficial effect on the SASP induced by TBHP in chondrocytes.

### 3.4 βOHB reduces TBHP‐induced oxidative stress and chondrocyte apoptosis

**FIGURE 2:**
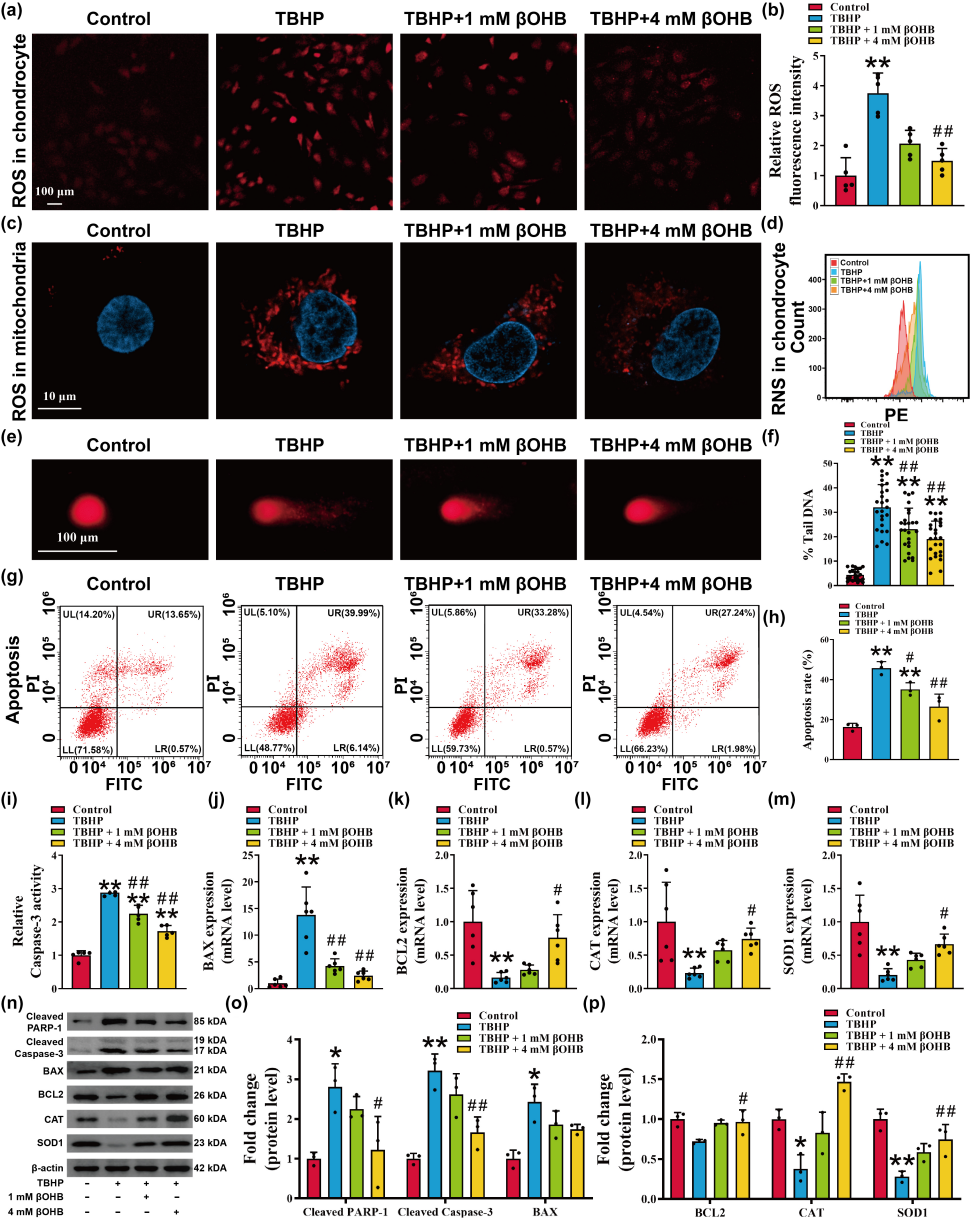
βOHB reduces TBHP‐induced oxidative stress and chondrocyte apoptosis. (a, b) ROS levels in chondrocytes were measured, with a scale bar of 100 μm, *n* = 5. (c) Representative images of mitochondria ROS were obtained through confocal microscopy, with a scale bar of 10 μm. (d) The reactive nitrogen species of chondrocytes was assessed using flow cytometry. (e) Representative images of the comet experiment were captured, with a scale bar of 100 μm. (f) Quantification of the tail DNA content (tail DNA intensity/cell DNA intensity) of the comet experiment, *n* = 25. (g, h) The apoptosis ratio of chondrocytes was assessed using flow cytometry, *n* = 3. (i) Caspase‐3 activity in total cell lysates was measured using the Caspase‐3 substrate, acetyl‐Asp‐Glu‐Val‐Asp. The activity was normalized to total protein concentration and presented as fold activity to the control group. (j–m) The mRNA expression levels of BAX, BCL2, CAT, and SOD1 were quantified using reverse transcription–quantitative polymerase chain reaction (RT‐qPCR), *n* = 6. (n–p) The protein expression levels of cleaved PARP‐1, cleaved Caspase‐3, BAX, BCL2, CAT, and SOD1 were analyzed using western blotting, *n* = 3. The obtained data were subjected to analysis using the one‐way ANOVA statistical method. *p < 0.05, **p < 0.01 versus the control group; ^#^ *p* < 0.05, ^##^ *p* < 0.01 versus the TBHP group.

It is well‐established that the accumulation of ROS plays a crucial role in chondrocyte senescence (Chen et al., 2023). Therefore, we further investigated the impact of βOHB on TBHP‐induced oxidative stress and apoptosis. Our results demonstrated that TBHP significantly increased the fluorescence intensity of ROS in both chondrocytes and mitochondria, whereas treatment with βOHB significantly reduced the intracellular accumulation of ROS in chondrocytes (Figure 2a–c). Furthermore, the flow cytometry analysis revealed that βOHB mitigated the increase in intracellular RNS induced by TBHP (Figure 2d).

Given that excessive oxidative stress has been linked to DNA damage and apoptosis (Arra et al., 2022), we investigated the impact of βOHB on apoptosis. The comet assay results demonstrated that TBHP led to observable tailing in cells, indicative of cellular DNA damage, whereas treatment with βOHB resulted in a reduction in comet tail length relative to the entire cell (Figure 2e, f, *p* < 0.01). The flow cytometry results demonstrated an increase in the apoptosis ratio of chondrocytes following TBHP intervention, which was subsequently reduced by βOHB treatment (Figure 2g, h, *p* < 0.05). Caspase‐3 is a widely expressed member of a conserved family of proteins, generally recognized for their activated proteolytic roles in the execution of apoptosis (Eskandari & Eaves, 2022). Our research also indicated that TBHP exposure heightened caspase‐3 activity in cells, whereas βOHB treatment led to a decrease in caspase‐3 activity (Figure 2i, *p* < 0.01). Moreover, the results obtained from RT‐qPCR and western blotting analyses demonstrated that TBHP caused a decrease in the mRNA and protein levels of CAT, SOD1, and BCL2, while simultaneously increasing the expression of cleaved Caspase‐3, cleaved PARP‐1, and BAX (Figure 2j–p, *p* < 0.05). Conversely, βOHB treatment reversed these alterations (Figure 2j–p, *p* < 0.05). These findings suggest that βOHB possesses a protective effect against TBHP‐induced oxidative stress and chondrocyte apoptosis.

### 3.5 βOHB improves mitophagy in TBHP‐induced chondrocytes

**FIGURE 3:**
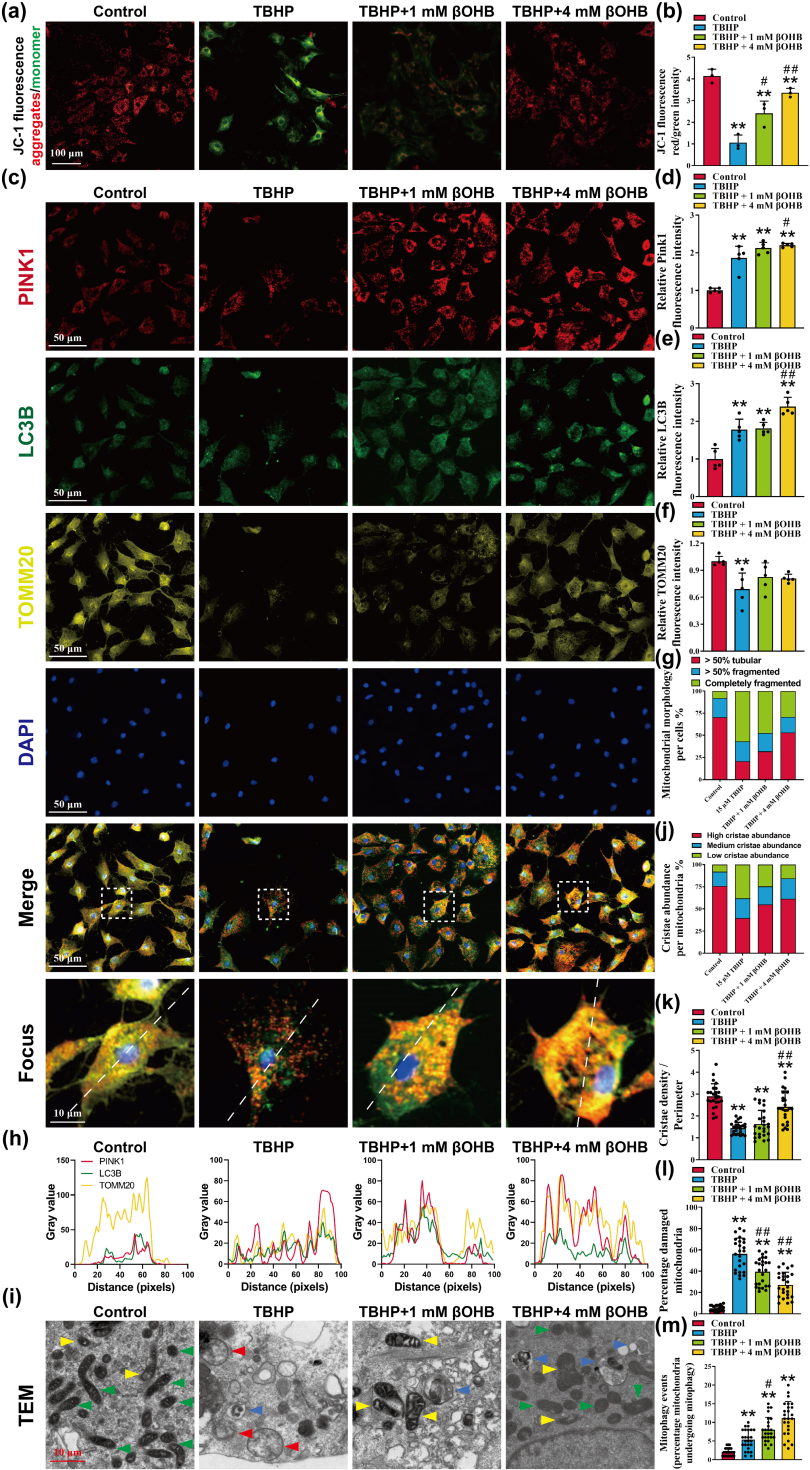
βOHB improves mitophagy in TBHP‐induced chondrocytes. (a, b) Representative images of mitochondrial membrane potential assay were obtained using JC‐1, with a scale bar of 100 μm, *n* = 3. (c–f) Representative images and relative fluorescence intensity of PINK1 (red), LC3B (green), TOMM20 (yellow), and cell nuclei (blue) immunofluorescence were captured, with a scale bar of 50 μm, *n* = 5. (g) The ratio of tubular mitochondria and fragmented mitochondrial was observed based on the immunofluorescence staining results of TOMM20, *n* = 25. (h) Co‐localization of PINK1 (red), LC3B (green), TOMM20 (yellow). (i) Microstructural detection of mitophagy by transmission electron microscopy and normal mitochondria with high cristae abundance (green arrow), mitochondria with medium cristae abundance (yellow arrow), damaged mitochondria with low cristae abundance (red arrow), and autophagosomes with mitochondrial‐like organelles (blue arrow) were as indicated, scale bar = 10 μm. (j) The cristae abundance in mitochondria was quantified by the images of transmission electron microscopy, *n* = 25. (k) The ratio of cristae density and perimeter in mitochondria was quantified by the images of transmission electron microscopy, *n* = 25. (l, m) The percentage of damaged mitochondria and mitophagy events were quantified by the images of transmission electron microscopy, *n* = 25. The obtained data were subjected to analysis using the one‐way ANOVA statistical method. *p < 0.05, **p < 0.01 versus the control group; ^#^ *p* < 0.05, ^##^ *p* < 0.01 versus the TBHP group.

The accumulation of dysfunctional mitochondria in senescent chondrocytes has been identified as the cause of increased levels of ROS within the cells. It has been observed that restoring mitochondrial function can effectively inhibit chondrocyte senescence (Shang et al., 2023). The measurement of Δψm serves as a sensitive indicator of mitochondrial function, and the fluorescent probe JC‐1 is commonly used to detect Δψm. Under normal physiological conditions, JC‐1 primarily exists in a polymer form, emitting red fluorescence. However, during the early stages of apoptosis, the reduction in Δψm causes JC‐1 to transition to a monomeric form, emitting green fluorescence. Consequently, a decrease in the red/green fluorescence ratio indicates mitochondrial depolarization and loss of membrane potential due to damage (Tao et al., 2024). In comparison to the control group, chondrocytes in the TBHP group exhibited a significant reduction in red light intensity and an increase in green light intensity, indicating a decrease in Δψm. Conversely, intervention with βOHB partially reversed the decrease in Δψm induced by TBHP (Figure 3a, b, *p* < 0.05).

Mitophagy, a process that selectively eliminates damaged or dysfunctional mitochondria through autophagic machinery, plays a crucial role in maintaining mitochondrial quality control and homeostasis (Sun et al., 2021). Our hypothesis posits that mitochondrial autophagy serves as a critical mechanism by which βOHB preserves mitochondrial membrane potential. The results of the immunofluorescence analysis indicated that TBHP induced upregulation of the intracellular mitochondrial autophagy markers LC3B and PINK1, while downregulating the expression of the outer mitochondrial membrane receptor TOMM20 (Figure 3c–f). TOMM20 is a protein located in the outer membrane of mitochondria, serving as a marker for mitochondrial quality (Konig et al., 2021). Analysis of TOMM20 morphology indicated that healthy cell mitochondria exhibited a consistent tubular form, whereas exposure to TBHP resulted in heightened mitochondrial fragmentation (Figure 3g). Furthermore, treatment with βOHB led to further enhancement of LC3B and PINK1 expression, as well as facilitated co‐localization of LC3B, PINK1 and TOMM20, indicating promotion of mitophagy by βOHB (Figure 3c–e, h). Analysis using transmission electron microscopy revealed damaged mitochondria with low cristae abundance following TBHP intervention, while treatment with βOHB resulted in improved mitochondrial structure and enhanced formation of autophagolysosomes with mitochondrial‐like organelles (Figure 3i). Our findings demonstrated that TBHP induced an upregulation in the proportion of damaged mitochondria characterized with low cristae abundance. Conversely, treatment with βOHB resulted in an increased proportion of mitochondria exhibiting high cristae abundance, an elevated cristae density‐to‐perimeter ratio, and an enhanced frequency of mitophagy events, while concurrently reducing the percentage of damaged mitochondria (Figure 3j–m).

**FIGURE 4:**
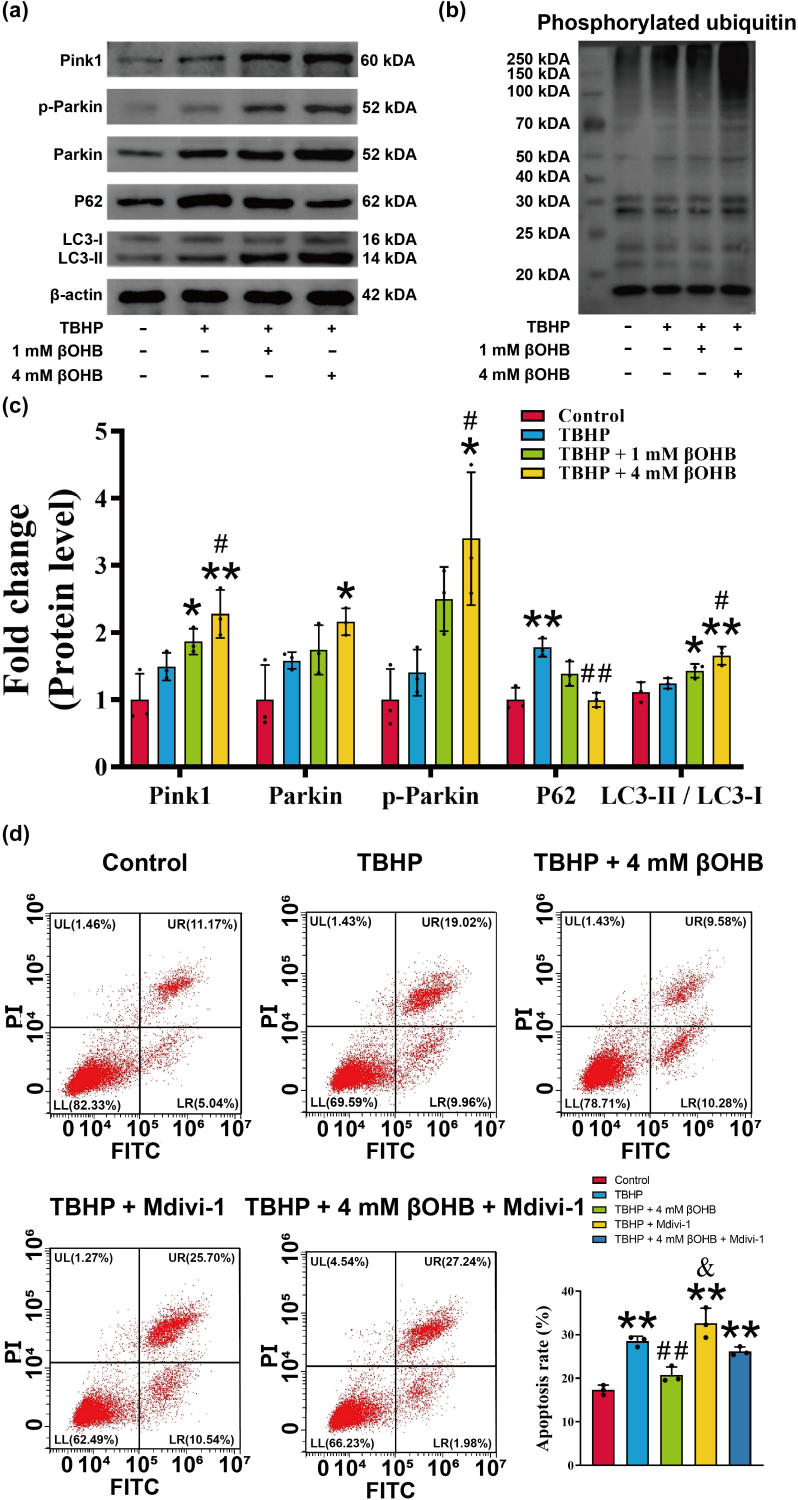
βOHB improves mitophagy in TBHP‐induced chondrocytes. (a) The protein expression levels of Pink1, p‐Parkin, Parkin, P62, and LC3B were assessed using western blotting, *n* = 3. (b) Phosphorylated ubiquitin was detected by western blotting. (c) The relative protein expression levels of Pink1, p‐Parkin, Parkin, P62, and LC3B were quantified by the ImageJ software (version 1.8). (d) The apoptosis ratio of chondrocytes was assessed using flow cytometry, *n* = 3. The obtained data were subjected to analysis using the one‐way ANOVA statistical method. *p < 0.05, **p < 0.01 versus the control group; ^#^ *p* < 0.05, ^##^ *p* < 0.01 versus the TBHP group; ^&^ *p* < 0.05 versus the TBHP + 4 mM group βOHB group.

Activation of PINK1/Parkin activation through phosphorylation of its ubiquitin and ubiquitin‐like domain by PINK1 plays a crucial role in inducing mitophagy (Niu et al., 2020). The western blotting analysis revealed that the control group exhibited low levels of mitophagy, as evidenced by the low expression of PINK1, p‐Parkin, Parkin, P62, LC3B II, and p‐Ub (Figure 4a–c). However, the intervention of TBHP slightly augmented the expression of PINK1, p‐Parkin, Parkin, LC3B II, and p‐Ub, indicating the initiation of mitophagy (Figure 4a–c, *p* > 0.05). Nevertheless, the accumulation of P62 indicated a disruption in autophagic flux within the TBHP group (Figure 4a, c, *p* < 0.05). Conversely, the administration of βOHB not only further enhanced the expression of PINK1, p‐Parkin, Parkin, LC3B II, and p‐Ub but also reduced the expression of P62, thereby indicating the restoration of cellular autophagy (Figure 4a–c, *p* < 0.05). Next, we investigated the impact of inhibiting mitophagy with Mdivi‐1 on apoptosis. Previous research demonstrated that 50 μM Mdivi‐1 effectively suppressed the expression of PINK1 and Parkin in chondrocytes (Liu et al., 2023). Therefore, we utilized the same concentration for our intervention. Flow cytometry analysis revealed a significant increase in apoptosis in chondrocytes following TBHP treatment. Interestingly, the addition of Mdivi‐1 prevented βOHB from reversing the detrimental effects of TBHP (Figure 4d, *p* < 0.05). These findings indicate that βOHB has the potential to mitigate chondrocyte apoptosis induced by TBHP through the promotion of mitophagy.

### 3.6 Supplementation of βOHB alleviated OA progression in rats

To further assess the impact of βOHB in vivo, an experimental model of rat OA was established through ACLT (Figure S3a). The intragastric administration of βOHB did not result in a significant change in body weight in rats (Figure S3b). However, it did lead to a significant increase in βOHB concentrations in both synovial fluid and serum (Figure S3c, d). Additionally, βOHB alleviated the levels of nitric oxide, TNF‐α, IL6, and PGE2 in the serum of OA rats (Figure S3e–h). Histological examination utilizing H & E and saffronin O‐fast green staining revealed that βOHB at doses of 100 or 200 mg/kg mitigated cartilage degradation and lowered OARSI scores (Figure S3i–k). These findings indicate that the intraperitoneal administration of βOHB effectively attenuated the progression in OA rats.

Immunohistochemistry results further demonstrated that βOHB promoted HCAR2, Col2a1, PINK1, Parkin expression, decreased IL6, MMP13, P16, P21, and P62 expression in chondrocytes (Figure S4). Hence, the aforementioned significant findings suggest that the activation of mitophagy by βOHB may serve as a potential therapeutic approach for alleviating OA.

### 3.7 βOHB exerts chondroprotective effects via HCAR2

**FIGURE 5:**
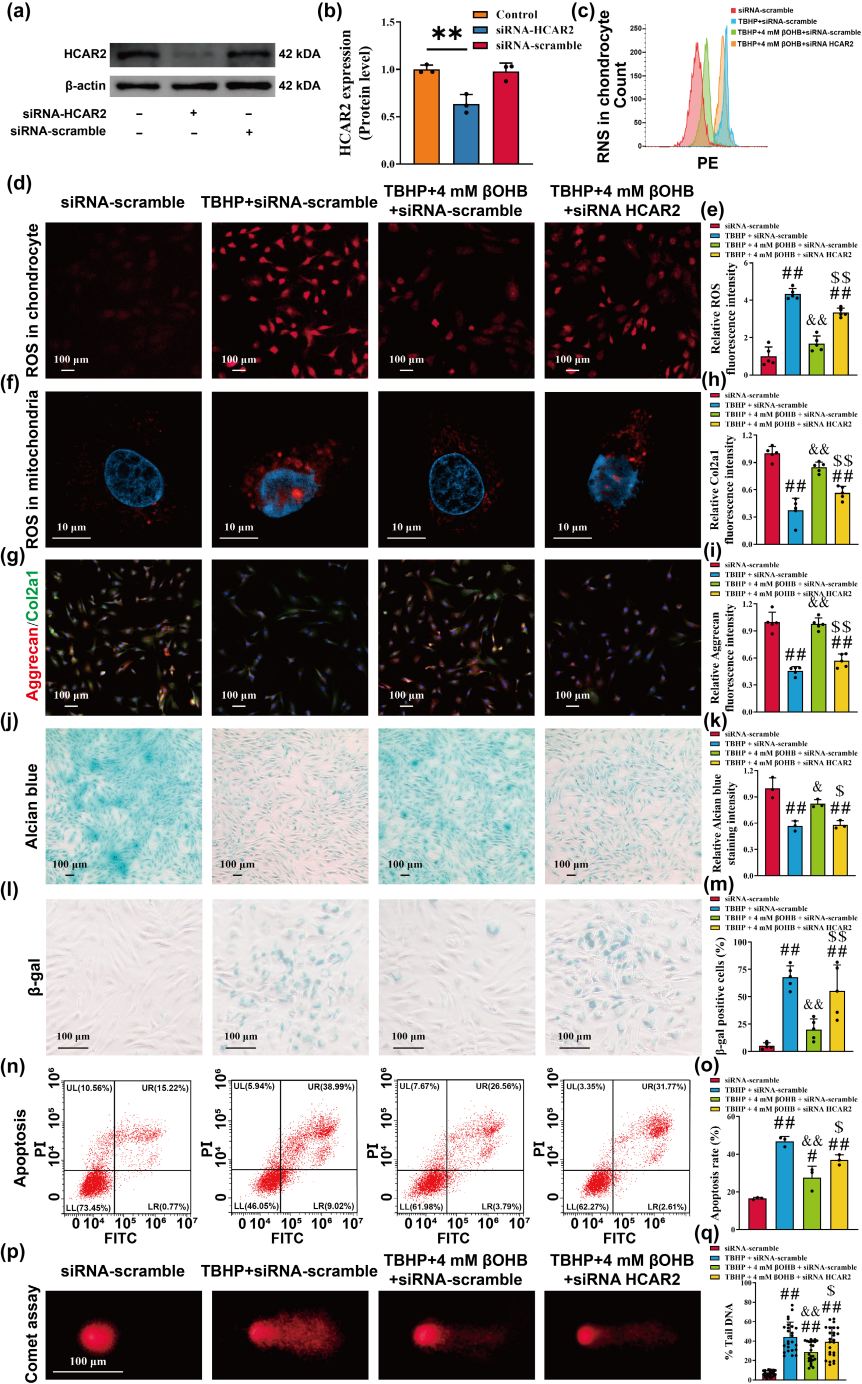
βOHB exerts chondroprotective effects via HCAR2 in TBHP‐induced chondrocytes. (a, b) The protein expression levels of HCAR2 were evaluated by western blotting, *n* = 3. (c) The reactive nitrogen species of chondrocytes was assessed using flow cytometry. (d, e) ROS levels in chondrocytes were measured, with a scale bar of 100 μm, *n* = 5. (f) Representative images of mitochondria ROS were obtained through confocal microscopy, with a scale bar of 10 μm. (g–i) Representative images and relative fluorescence intensity of Aggrecan (red), Col2a1 (green), and cell nuclei (blue) immunofluorescence were captured, with a scale bar of 100 μm, *n* = 5. (j, k) Representative images of Alcian Blue staining were obtained, with a scale bar of 100 μm. (l, mM) Senescence‐associated β‐galactosidase (SA‐β‐gal) staining was utilized to observe the aging phenotype, with a scale bar of 100 μm, *n* = 5. (n, o) The apoptosis ratio of chondrocytes was assessed using flow cytometry, *n* = 3. (p) Representative images of the comet experiment were captured, with a scale bar of 100 μm. (q) Quantification of the tail DNA content (tail DNA intensity/cell DNA intensity) of the comet experiment, *n* = 25. The obtained data were subjected to analysis using the one‐way ANOVA statistical method. **p < 0.01 versus the control group; ^#^ *p* < 0.05, ^##^ *p* < 0.01 versus the siRNA‐scramble group; ^&^ *p* < 0.05, ^&&^ *p* < 0.01 versus the TBHP + siRNA‐scramble group; ^$^ *p* < 0.05, ^$$^ *p* < 0.01 versus the TBHP + 4 mM βOHB + siRNA‐scramble group.

As a high‐affinity receptor for βOHB, HCAR2 has been scientifically established to play a crucial role in maintaining normal Δψm in M2 macrophages (Chen et al., 2018). Therefore, we employed siRNA to suppress the expression of HCAR2 in chondrocytes and investigated whether the chondroprotective efficacy of βOHB relied on HCAR2 (Figure 5a, b, *p* < 0.01).

Flow cytometry results demonstrated that βOHB mitigated the accumulation of RNS in chondrocytes. Besides, βOHB reduced the accumulation of ROS in chondrocytes and mitochondria (Figure 5c–f). Immunofluorescence staining results showed that βOHB restored the expression of Col2a1 and Aggrecan in TBHP‐treated chondrocytes (Figure 5g–i, *p* < 0.01). However, this phenomenon was not observed in chondrocytes with HCAR2 knockdown. In addition, Alcian blue staining demonstrated that the administration of βOHB did not mitigate the detrimental consequences of TBHP after the suppression of HCAR2 (Figure 5j, k, *p* < 0.01). The above results showed that βOHB was unable to reverse the phenotypic loss of chondrocytes caused by TBHP intervention in HCAR2 knockdown chondrocytes.

The SA‐β‐Gal staining demonstrated that βOHB reversed the cellular senescence induced by TBHP in chondrocytes, but this effect was abolished following HCAR2 knockdown (Figure 5l, m, *p* < 0.01). Flow cytometry results indicated a significant reduction in the apoptosis ratio of chondrocytes following treatment with βOHB, but the knockdown of HCAR2 resulted in an increased apoptosis ratio (Figure 5n, o, *p* < 0.05). The comet assay demonstrated that in HCAR2 knockdown chondrocytes, βOHB was unable to mitigate TBHP‐induced DNA damage (Figure 5p, q, *p* < 0.05). The above findings indicate that βOHB is ineffective in mitigating TBHP‐induced chondrocyte oxidative stress damage and apoptosis after HCAR2 knockdown.

JC‐1 results indicated that βOHB augmented the ratio of red fluorescence to green fluorescence, implying a reduction in the number of cells undergoing early apoptosis, while this effect of βOHB was nullified upon the inhibition of HCAR2 (Figure S5a, b, *p* < 0.01). Immunofluorescence analysis revealed that HCAR2 knockdown resulted in decreased LC3B and PINK1 expression, enhanced mitochondrial fragmentation, and hindered co‐localization of LC3B, PINK1, and TOMM20 (Figure S5c–h). Transmission electron microscopy findings indicated that βOHB did not restore mitochondrial cristae abundance in HCAR2 knockdown chondrocytes, leading to an increase in damaged mitochondria and a reduction in mitochondrial autophagy events (Figure S5i–m).

RT‐qPCR results revealed that βOHB downregulated the RNA expression of P16, P21, IL6, MMP13, and BAX, while upregulating the expression of Col2a1, Aggrecan, BCL2, CAT, and SOD1 in TBHP‐stimulated chondrocytes, but the effects of βOHB diminished in chondrocytes with HCAR2 knockdown (Figure S6a, b, *p* < 0.05). The western blotting results revealed that βOHB significantly upregulated the expression of P16, P21, iNOS, IL6, MMP13, Cleaved PARP‐1, Cleaved Caspase‐3, BAX, PINK1, p‐Parkin, Parkin, and LC3B II, and promoted the phosphorylation of ubiquitin, while downregulating the expression of BCL2, CAT, SOD1, and P62. Knockdown of HCAR2 resulted in suppressed expression of PINK1, Parkin, and LC3B II, and elevated expression of P62, indicating impaired mitophagy in chondrocytes (Figure S6c–e, *p* < 0.05). These results offer evidence for the beneficial effects of βOHB in mitigating TBHP‐induced oxidative stress, apoptosis, and mitophagy in chondrocytes through the involvement of HCAR2.

### 3.8 Effects of βOHB on mitochondrial respiration and oxidative phosphorylation in chondrocytes

Mitochondria play a crucial role in meeting the energy demands of cells through the production of ATP. OA may arise when there is a disruption in energy metabolism leading to decreased ATP synthesis in chondrocytes (Chen et al., 2022). βOHB serves as a vital metabolic fuel that supports bioenergetic metabolism, particularly during periods of nutrient deprivation (Puchalska & Crawford, 2021). In tissues outside of the liver, βOHB is converted to acetyl‐CoA and enters the tricarboxylic acid cycle through a series of enzymatic reactions known as ketolysis (Luda et al., 2023). Exposure of activated CD8 Teff cells to βOHB for a short duration (2 h) resulted in a modest increase in their basal OCR and ATP production through oxidative phosphorylation (Luda et al., 2023). In order to assess the impact of βOHB on energy metabolism in chondrocytes, real‐time evaluation of mitochondrial function was conducted using the Seahorse Bioscience XFp extracellular flux analyzer under different stimuli.

Basal respiration, maximal respiration, and spare respiratory capacity are intricately linked to cell viability (Dhingra & Kirshenbaum, 2015; Huang et al., 2019). Treatment with βOHB resulted in a slight increase in maximal respiration and spare respiratory capacity of chondrocytes in the absence of TBHP intervention compared to the control group (Figure S7a–d, *p* > 0.05). However, this difference did not reach statistical significance. TBHP intervention led to a decrease in basal respiration, maximal respiration, and spare respiratory capacity compared to the control group, indicating a reduction in ATP production capacity and severe mitochondrial dysfunction in chondrocytes (Figure S7a–d, *p* < 0.05). Meanwhile, a decrease in chondrocyte respiration was observed in response to the heightened oxidizing environment. Conversely, treatment with βOHB demonstrated the ability to mitigate mitochondrial oxidative damage by enhancing spare respiratory capacity, maximal respiration, and ATP production capacity, indicating its potential for mitigating such damage (Figure S7a–f, *p* < 0.05). Furthermore, a mild reduction in chondrocyte respiration was noted following HCAR2 knockdown, although this change did not reach statistical significance (Figure S7a–f, *p* > 0.05). Nevertheless, the therapeutic efficacy of βOHB against TBHP‐induced damage was negated upon HCAR2 knockdown (Figure S7a–f, *p* < 0.05). The above findings suggest that βOHB regulates chondrocyte energy metabolism in chondrocytes subjected to oxidative stress.

### 3.9 βOHB exerts chondroprotective effects through phosphorylation of AMPK

AMPK is a heterotrimeric complex consisting of one catalytic subunit α and two regulatory subunits β and γ, and is closely associated with energy metabolism (Yao et al., 2023). βOHB has been shown to activate the HCAR2/AMPK axis, leading to prevention of hepatic steatosis and the regulation of neutrophil‐mediated inflammation (Carretta et al., 2020; Lee et al., 2020). Activation of AMPK can modulate cell metabolism through PINK1‐PRKN‐dependent mitophagy, thereby improving renal oxidative stress and renal tubulointerstitial fibrosis in diabetic mice (Gong et al., 2022; Han et al., 2021). This suggests that βOHB may play a protective role in chondrocytes by targeting the HCAR2/AMPK/PINK1 axis.

**FIGURE 6:**
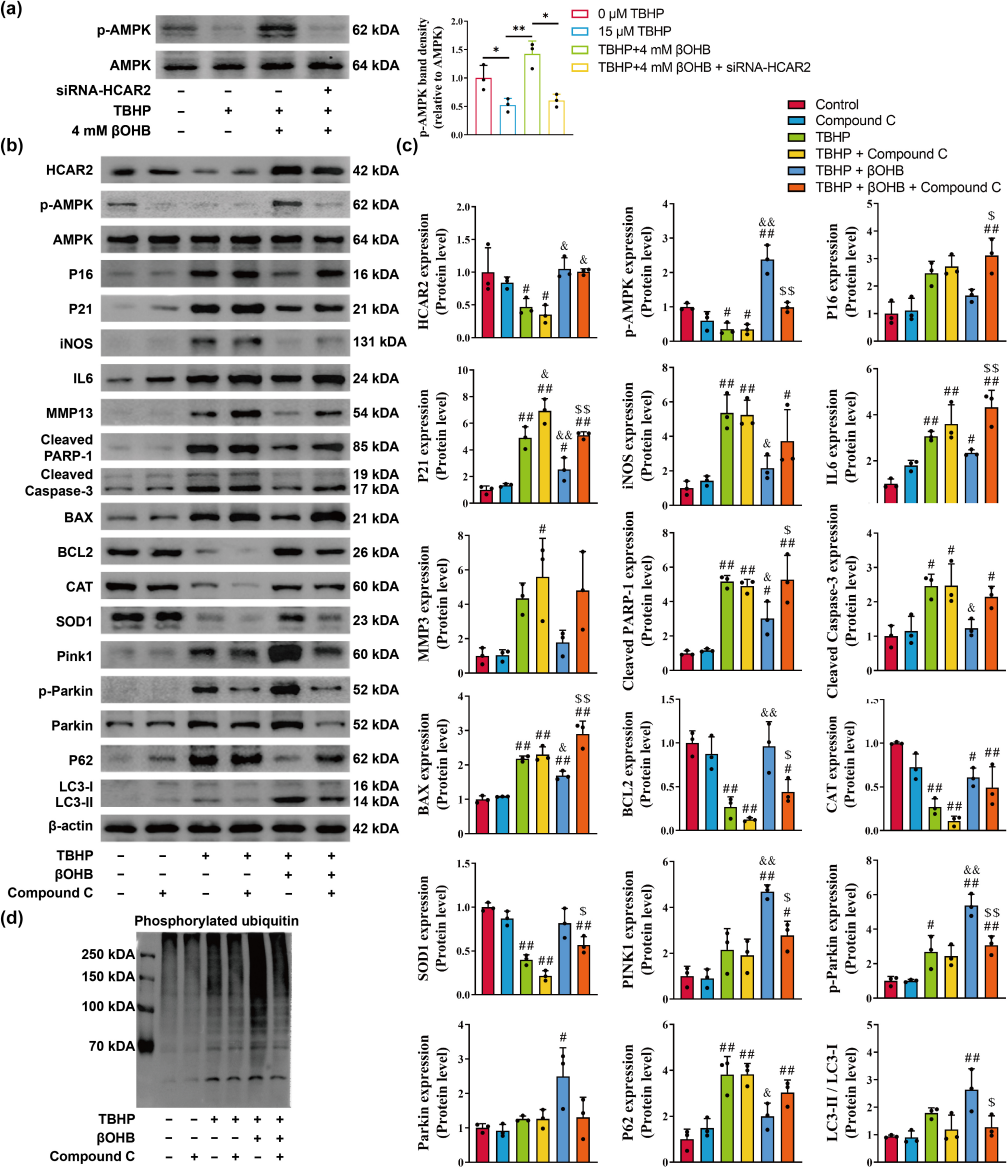
βOHB exerts chondroprotective effects through activation of AMPK. (a) The protein expression level of p‐AMPK was assessed using western blotting, *n* = 3. (b) The protein expression levels of HCAR2, p‐AMPK, AMPK, P16, P21, iNOS, IL6, MMP13, Cleaved PARP‐1, Cleaved caspase‐3, BAX, BCL2, CAT, SOD1, Pink1, p‐Parkin, Parkin, P62, and LC3B were assessed using western blotting, *n* = 3. (c) The relative protein expression levels of HCAR2, p‐AMPK, AMPK, P16, P21, iNOS, IL6, MMP13, Cleaved PARP‐1, Cleaved caspase‐3, BAX, BCL2, CAT, SOD1, Pink1, p‐Parkin, Parkin, P62, and LC3B were quantified by the ImageJ software (version 1.8). (d) Phosphorylated ubiquitin was detected by western blotting. The obtained data were subjected to analysis using the one‐way ANOVA statistical method. *p < 0.05, **p < 0.01; ^#^ *p* < 0.05, ^##^ *p* < 0.01 versus the Control group; ^&^ *p* < 0.05, ^&&^ *p* < 0.01, versus the TBHP group; ^$^ *p* < 0.05, ^$$^ *p* < 0.01 versus the TBHP + βOHB group.

The results of the western blot analysis indicated that intervention with TBHP led to a decrease in AMPK phosphorylation, while βOHB significantly increased AMPK phosphorylation in a manner dependent on HCAR2 (Figure 6a, *p* < 0.05). Subsequently, the AMPK antagonist Compound C (25 μM) was utilized to further investigate the potential mechanisms. The western blot analysis revealed that inhibition of AMPK did not impact the upregulation of HCAR2 expression by βOHB intervention (Figure 6b, c). However, Compound C promoted SASP, oxidative stress and apoptotic phenotypes in chondrocytes (Figure 6b, c). Moreover, the expression of PINK1, Parkin, p‐Parkin, and p‐Ub was suppressed with Compound C, suggesting inhibition of mitophagy (Figure 6b–d). These findings suggest that βOHB exerts chondroprotective effects through the phosphorylation of AMPK.

### 3.10 βOHB exerts protective effects on OA rats through the HCAR2/AMPK/PINK1 pathway

To investigate the therapeutic mechanism of βOHB, mepenzolate bromide (HCAR2 inhibitor), Compound C (AMPK antagonist), and Mdivi‐1 (mitochondrial division inhibitor) were injected into the knee joints of rats. H & E staining and safranin O‐fast green staining results indicated that βOHB was unable to reverse the damage caused by ACLT after injection of the inhibitors (Figure S8a–c, *p* < 0.05). Immunohistochemical staining showed that ACLT decreased the expression of HCAR2 and p‐AMPK, while treatment with βOHB increased their expression (Figure S8d–i, *p* < 0.05). Additionally, injection of mepenzolate bromide counteracted the pro‐expression effects of βOHB on HCAR2, p‐AMPK, PINK1, p‐Parkin, and Parkin (Figure S8d–i, *p* < 0.05). Compound C injection did not suppress HCAR2 expression but did inhibit p‐AMPK levels and mitophagy (Figure S8d–i, *p* <0.05). In contrast, inhibition of mitophagy in rat cartilage tissue by Mdivi‐1 injection did not alter the expression of HCAR2 and p‐AMPK (Figure S8d–i, *p* < 0.05). These findings support the conclusion that βOHB delays OA progression through the HCAR2/AMPK/PINK1 signaling pathway.

## 4 DISCUSSION

OA is one of the predominant causes of disability in the elderly population. Regrettably, existing pharmaceutical interventions merely offer transient relief from OA pain symptoms, necessitating further exploration of drugs capable of impeding the pathogenesis of OA. TBHP, a well‐established inducer of free radical reactions, is extensively employed in creating cellular models for oxidative stress and senescence (Jiang et al., 2022; Pan et al., 2023; Shang et al., 2023). While KD has demonstrated significant potential in suppressing inflammation and premature aging, it is important to acknowledge that KD may also elevate the likelihood of various disorders and diseases, such as kidney disease, fatigue, headache, and dizziness, thereby potentially impacting the health of patients, particularly the elderly who constitute the majority of the OA‐affected population (Dynka et al., 2023; Ferrer et al., 2023; Gorini et al., 2023). Consequently, it becomes imperative to investigate alternative treatment strategies that are more viable.

Ketone bodies play a crucial role as an energy source in biological processes, with βOHB constituting approximately 70% of the overall circulating ketone bodies (Puchalska & Crawford, 2017). During periods of starvation, the concentration of βOHB in the bloodstream can rise to 8 mM, thereby becoming the primary source of energy for the body (Ji et al., 2022; Shimazu et al., 2013). Through synovial metabolomics analysis, it has been determined that βOHB serves as a significant biomarker for synovial inflammation in patients with OA (Mickiewicz et al., 2015). Recent findings have revealed that ketone bodies possess not only energy‐carrying capabilities but also signaling functions (Newman & Verdin, 2014). This study demonstrated that both KD feeding and intraperitoneal injection of βOHB effectively mitigated disease progression in rats with OA. Additionally, βOHB exhibited the ability to reverse oxidative stress, apoptosis, and senescence induced by TBHP in chondrocytes, while maintaining the equilibrium between extracellular matrix synthesis and degradation. These findings suggest that βOHB shows promise as a potential therapeutic target for OA.

Chondrocytes, which exclusively constitute articular cartilage, play a crucial role in extracellular matrix synthesis by continuously secreting collagen type II and Aggrecan (Guilak et al., 2018). Type II collagen and Aggrecan are widely recognized as the predominant cartilage proteins and are commonly utilized as classical biomarkers for OA (Bay‐Jensen et al., 2022). Type II collagen, the primary protein found in cartilage, functions as a fibrillar collagen that provides structural support to the extracellular matrix of cartilage (Eyre, 2002). Aggrecan serves as the principal glycoprotein within the extracellular matrix of articular cartilage (Heinegard & Saxne, 2011). Aggrecan functions as the predominant glycoprotein in the extracellular matrix of articular cartilage, binding to hyaluronic acid and link proteins to attract cations and osmotically obligated water into the extracellular matrix (Kiani et al., 2002). This process results in the formation of a hydrated gel that imparts compressive strength to the cartilage. The degradation of type II collagen and Aggrecan in articular cartilage by matrix metalloproteinases is recognized as a significant pathological characteristic of OA (Zhang et al., 2009). MMP‐13 is also acknowledged as a marker of terminal chondrocyte differentiation (Inada et al., 2004). Our findings indicate that treatment with βOHB leads to the downregulation of MMP13 expression and the promotion of col2a1 and Aggrecan expression, suggesting that βOHB may contribute to the maintenance of a healthy chondrocyte phenotype rather than a hypertrophic one.

Oxidative stress, characterized by an imbalance between oxidative and antioxidant activities, is recognized as a significant factor in aging and disease, and is implicated in the pathophysiology of OA (Zhuang et al., 2018). To induce the production of ROS and RNS in cells, we utilized TBHP to induce the production of iNOS. iNOS serves as a pivotal enzyme in the synthesis of nitric oxide and the sustained activity of nitric oxide can lead to the inhibition of extracellular matrix secretion in chondrocytes (Ahmad et al., 2020). This disruption in extracellular matrix balance within the cartilage microenvironment ultimately results in the quality of cartilage deteriorates (Santoro et al., 2015). After experiencing injury, such as oxidative stress, chondrocytes can maintain the balance of cartilage through adaptive cell proliferation. However, chondrocytes that re‐enter the cell division cycle in this manner are more prone to cellular senescence, which contributes to the progression of OA (Coryell et al., 2021). Senescent chondrocytes not only display a portion of chondrocytes that are positive for SA‐β‐Gal and increased levels of P16 and P21, but also release inflammatory factors and enzymes that degrade the extracellular matrix (Tsuchida et al., 2014). The upregulation of SASP factors initiates an inflammatory response and senescence in neighboring healthy cells, ultimately resulting in extracellular matrix degradation and cartilage degeneration (Jeon et al., 2018). Mitigating chondrocyte senescence diminishes the production of MMPs and pro‐inflammatory factors, thereby preserving the structural integrity of articular cartilage (Xie et al., 2021). Activation of the cell death program occurs when cellular stress surpasses a critical threshold in intensity or duration. Upon initiation of the apoptotic program, caspases are activated to cleave a diverse array of proteins in order to facilitate apoptosis. Caspase‐3, a member of the Cysteine‐ASPartic proteASES family renowned for their role in mediating the specific cleavage of substrates during apoptosis, is responsible for cleaving various downstream proteins, ultimately leading to the characteristic morphological changes observed in apoptotic cells (Eskandari & Eaves, 2022). Our findings indicate that the cleavage of PARP‐1 by activated caspase‐3 results in PARP‐1 inactivation and subsequent inhibition of DNA repair capabilities, as evidenced by a notable increase in tail DNA percentage in the comet experiment.

Although βOHB has been reported to inhibit oxidative stress in various studies, its role in OA remains inadequately explored (Kawakami et al., 2022; Luo et al., 2022; Miyauchi et al., 2019). Our findings demonstrate that βOHB significantly attenuates the expression of senescence markers (p16, p21, SA‐β‐Gal), inflammatory factors (IL6, iNOS), apoptotic markers (BAX, cleaved caspase‐3, cleaved PARP‐1), and promotes the synthesis of extracellular matrix (Aggrecan and Col2a1), thereby enhancing chondrocyte resilience against oxidative stress induced by TBHP.

The articular cartilage lacks blood vessels and instead receives nutrients and oxygen through the diffusion of synovial fluid and substances from the subchondral bone (Milner et al., 2006). The oxygen concentration at the surface of the joint is approximately 6%, while it decreases to 1% in the deeper layers of the cartilage, indicating a consistently low oxygen environment (Shimomura et al., 2022). Chondrocytes affected by OA frequently demonstrate the release of pro‐inflammatory cytokines, damage from oxidative stress, impaired mitochondrial energy metabolism, and imbalances in protein metabolism, all of which significantly impact the physiological function of the cartilage tissue (Xu et al., 2020). While normal cartilage metabolism primarily occurs in the absence of oxygen, joints affected by OA commonly exhibit mitochondrial dysfunction (Blanco & Rego‐Perez, 2020). Cellular autophagy is the process by which intracellular proteins and organelles are degraded by lysosomes. Mitophagy, a specific form of cellular autophagy, is responsible for the removal of dysfunctional mitochondria and serves as a vital regulatory mechanism for maintaining cellular homeostasis. Mitophagy plays a pivotal role in preventing the generation of ROS and mitochondrial dysfunction, thereby aiding chondrocytes in their survival during pathological circumstances (Fernandez‐Moreno et al., 2022). Mitophagy dysfunction is observed in various age‐related ailments, and interventions aimed at enhancing mitophagy have exhibited encouraging outcomes in the context of muscle senescence, cardiovascular disorders, and neurological degenerative conditions (Palikaras et al., 2017). Consequently, the exploration of mitophagy targeting is deemed an efficacious strategy for addressing OA.

βOHB has been identified as a high‐affinity ligand for HCAR2, although the specific role of HCAR2 in chondrocytes remains unknown (Rahman et al., 2014). HCAR2 is a G protein‐coupled receptor widely expressed on cell membranes. The activation of HCAR2 by βOHB and nicotinic acid, leading to anti‐inflammatory effects, presents an intriguing potential for targeting this receptor in the treatment of gastrointestinal, cardiovascular, and neurogenic inflammatory disorders (Chen et al., 2018; Dmitrieva‐Posocco et al., 2022; Kaye et al., 2020; Moutinho et al., 2022; Singh et al., 2014). To the best of our knowledge, there is a lack of research on the role of HCAR2 in chondrocytes. Despite the strong agonist activity of nicotinic acid as a small molecule carboxylic acid, the side effects of cutaneous flushing pose a challenge to patient compliance (Walters et al., 2009). Therefore, our study focuses on the potential role of βOHB and its receptor HCAR2 in OA. The unanticipated necessity of HCAR2 in the anti‐oxidative stress properties of βOHB is noteworthy, considering that βOHB has previously demonstrated anti‐inflammatory effects by directly inhibiting inflammasome activation, irrespective of its known activation of HCAR2 (Y. Chen et al., 2018). Our study found that βOHB failed to reverse the damaging effects of TBHP on HCAR2 knockdown chondrocytes, suggesting that the therapeutic effect of βOHB is dependent on HCAR2.

The PINK1/Parkin pathway is widely recognized as a significant mechanism of mitophagy (Onishi et al., 2021). In normal circumstances, PINK1 relocates to the inner mitochondrial membrane in a manner contingent upon membrane potential, followed by degradation to maintain minimal levels (Yamano & Youle, 2013). When the mitochondrial transmembrane potential is disrupted, PINK1 accumulates and facilitates the recruitment and activation of Parkin in the cytoplasm, thereby promoting mitophagy for the elimination of impaired mitochondria (Geisler et al., 2010; Koyano et al., 2014). Parkin, an E3 ubiquitin ligase, exhibits robust expression in various tissues, implying a diverse array of physiological functions (Suen et al., 2010). The initiation of mitophagy through parkin recruitment is believed to entail the ubiquitylation of mitochondrial substrates by parkin, with its E3 ubiquitin ligase activity heightened upon translocation to mitochondria (Matsuda et al., 2010). Activation of PINK1‐dependent mitophagy through the use of suberoylanilide hydroxamic acid results in a reduction of TOMM20, heat shock protein 60, and translocase of the inner membrane 23 protein levels (Sun et al., 2022). This process is also associated with an increase in LC3 protein levels (autophagosomal marker), and a decrease in P62 protein levels (autophagic substrate) (Sun et al., 2022). LC3 (also called MAP1LC3 or LC3B, the ortholog of yeast ATG8) undergoes synthesis in the pro‐LC3 form, subsequent cleavage by ATG4B to generate LC3‐I, and conjugation in ubiquitin‐like reactions to phosphatidyl ethanolamine resulting in the formation of LC3‐II. The localization of LC3‐II to isolation membranes is essential for the elongation and closure of these membranes, ultimately leading to the formation of mature autophagosomes (Ashrafi & Schwarz, 2013). The accumulation of LC3‐II is indicative of a late stage in the autophagy pathway and is dependent on various protein complexes crucial for mitophagy (Ashrafi & Schwarz, 2013). During the process of mitophagy, the accumulation of the ubiquitin‐binding articulin protein P62 on depolarized mitochondria aids in the recruitment of damaged mitochondria to the autophagosome by binding to LC3 (Youle & Narendra, 2011). Conversely, the rapid accumulation of P62 following irreversible inflammation or oxidative stress implies the inhibition of mitophagy (Chen et al., 2020).

The involvement of mitophagy and associated proteins in OA remains a topic of debate (Sun et al., 2021). Chondrocytes stimulated with IL1β demonstrate characteristics of OA and an increase in mitophagy mediated by PRKN (Ansari et al., 2018). Depletion of PRKN results in impaired mitophagy, leading to mitochondrial dysfunction and oxidative stress (Ansari et al., 2018). Treatment with metformin activates the SIRT3‐PINK1‐PRKN signaling pathway, reversing IL1β‐induced oxidative stress in chondrocytes (C. Wang et al., 2019). These findings underscore the significance of the PINK1‐PRKN signaling pathway in regulating mitophagy and maintaining chondrocyte homeostasis in pathological conditions. In contrast, Shin et al. demonstrated that PINK1‐mediated mitophagy led to mitochondrial fragmentation and cell death in human chondrocytes and rats following monosodium iodoacetate treatment (Shin et al., 2019). PINK1 knockout mice exhibited decreased cartilage damage and pain behavior in OA rats (Shin et al., 2019). These results indicate a dual impact of mitophagy on cellular survival and functionality, potentially influenced by varying levels of mitophagy in pathological states. Intriguingly, our study reveals that βOHB reduces P62 expression, enhances the expression of PINK1 and Parkin, and elevates the LC3B I/II ratio, indicating the restoration of mitophagy. In summary, our research findings demonstrate that TBHP induces mitochondrial dysfunction and that βOHB restores mitophagy in an HCAR2‐dependent manner.

AMPK plays a crucial role in regulating cellular energy balance by promoting ATP production and inhibiting ATP consumption through the modulation of metabolic enzymes (Yao et al., 2023). Activation of the AMPK complex occurs when cellular energy levels are low, as indicated by the phosphorylation of Thr‐172 in the α‐subunit. AMPK expression and AMPKα phosphorylation are highly expressed in healthy articular cartilage. However, a significant decrease in AMPKα phosphorylation at the T172 locus was observed in articular cartilage from OA patients and surgery‐induced OA mice, suggesting that AMPK activation serves as a protective mechanism for articular cartilage (Petursson et al., 2013). The mechanisms underlying the reduction in AMPK expression and activation remain unclear (Yao et al., 2023). Previous studies have established a link between mitochondrial dysfunction in OA chondrocytes and aberrant AMPK activity (Wang et al., 2015). Our investigation revealed that treatment with TBHP decreased the phosphorylation of AMPK in chondrocytes, while an ACLT‐induced OA rat model also resulted in reduced AMPK phosphorylation. This impairment in AMPK activity subsequently led to mitochondrial dysfunction, resulting in inadequate energy production and increased ROS generation (Petursson et al., 2013). Research has indicated that the upregulation of total and phosphorylated AMPK expression through metformin administration can mitigate inflammation and postpone the onset of OA (Feng et al., 2020; Li et al., 2020; Wang et al., 2020). Furthermore, AMPK activation triggers essential kinases that facilitate autophagy, and metformin treatment enhances the expression of LC3 in chondrocytes (Feng et al., 2020; Guo et al., 2022; Hwang et al., 2021). Our investigation illustrated that βOHB elevates the phosphorylation status of AMPK both in vitro and in vivo, stimulating mitophagy and subsequently mitigating damage to chondrocytes in OA.

Additionally, we investigated the potential mechanisms of mepenzolate bromide (an HCAR2 inhibitor), Compound C (an AMPK antagonist), and Mdivi‐1 (a mitochondrial division inhibitor) in OA rat models. In our research, we observed that 200 mg/kg βOHB exhibited a capacity to impede the progression of OA in rats. However, the introduction of mepenzolate bromide, Compound C, and Mdivi‐1 significantly diminished the effectiveness of βOHB supplementation. Furthermore, mepenzolate bromide hindered the activation of AMPK and PINK1 activation by βOHB, while Compound C and Mdivi‐1 did not inhibit the upregulation of HCAR2 expression by βOHB. Compound C impeded the activation of PINK1 by βOHB, whereas Mdivi‐1 did not suppress the activation of AMPK induced by βOHB. Our findings suggest that βOHB may exert chondroprotective effects through the HCAR2/AMPK/PINK1 axis in rats.

However, it is important to acknowledge several limitations in this study. Firstly, due to the unavailability of healthy human chondrocyte resources, we utilized chondrocytes from rats. Secondly, the etiology of OA is multifaceted, and various modalities such as TBHP, IL‐1β, H_2_O_2_, continuous passaging, UV irradiation, hypoxia, mechanical overload, TGF‐β1, and FGF have been employed to establish in vitro chondrocyte senescence models (Rim et al., 2020; Xie et al., 2021). Thirdly, only wild‐type rats were used in this study. The utilization of HCAR2 knockout rats in future studies would enhance the validation of βOHB as a therapeutic strategy. Nevertheless, the extent to which these models accurately represent the in vivo situation of OA remains unknown.

## 5 CONCLUSION

The present study presents novel findings indicating that βOHB exhibits the ability to counteract TBHP‐induced chondrocyte inflammation, oxidative stress, senescence, and apoptosis, while also facilitating mitophagy via the HCAR2/AMPK/PINK1/Parkin pathway. Furthermore, both KD and intraperitoneal administration of βOHB demonstrate the potential to impede the advancement of OA in rats. These encouraging results contribute fresh insights into the clinical application of KD and βOHB supplementation for the treatment of OA.

## AUTHOR CONTRIBUTIONS

**Huangming Zhuang**: conceptualization, writing—original draft. Xunshan Ren: methodology, writing—review and editing. **Yuelong Zhang**: formal analysis, validation. **Huajie Li**: methodology. **Panghu Zhou**: conceptualization, project administration, funding acquisition.

## FUNDING INFORMATION

This work was supported by the National Natural Science Foundation of China (No. 82372489), the Hubei Medical Youth Tip‐Top Talent to Panghu zhou (No. CZ2024020005‐11), the Fundamental Research Funds for the Central Universities (No. 2042023kf0224), Wuhan University Education and Development Foundation (No. 2002330) and the Cross‐Innovation Talent Program of Renmin Hospital of Wuhan University (No. JCRCFZ‐2022‐019).

## CONFLICT OF INTEREST STATEMENT

The authors have declared that no competing interest exists.

## Supporting information


**Figure S1.**Effect of βOHB on chondrocyte viability. (A, B) The cell counting kit‐8 assay was employed to measure the toxic effects of different concentrations of βOHB and TBHP on chondrocytes, *n* = 3. (C, D) SA‐β‐gal staining was utilized to observe the aging phenotype, with a scale bar of 100 μm, *n* = 3. (E) The mRNA expression of P16 and P21 was evaluated using RT‐qPCR, *n* = 6. (F) The toxic effects of 15 μM TBHP and varying concentrations of βOHB on chondrocytes were measured by cell counting kit‐8 assay, *n* = 3. The obtained data was subjected to analysis using the one‐way ANOVA statistical method. *p < 0.05, **p < 0.01 versus the control group; ^#^ *p* < 0.05, ^##^ *p* < 0.01 versus the 15 μM TBHP group.

**Figure S2.**βOHB attenuates TBHP‐induced senescence‐associated secretory phenotype in chondrocytes. (A, B) SA‐β‐gal staining was utilized to demonstrate the ratio of senescence chondrocytes, with a scale bar of 100 μm, *n* = 5. (C, D) Representative images of Alcian Blue staining were obtained, with a scale bar of 500 μm. (E–G) Representative images of Aggrecan (red), Col2a1 (green), and cell nuclei (blue) immunofluorescence were captured, with a scale bar of 100 μm, *n* = 5. (H–J) The mRNA expression of Aggrecan, Col2a1, P16, P21, IL6, and MMP13 was evaluated using RT‐qPCR, *n* = 6. (K, L) The protein expression of P16, P21, iNOS, IL6, and MMP13 was evaluated through western blotting, *n* = 3. The obtained data were subjected to analysis using the one‐way ANOVA statistical method. *p < 0.05, **p < 0.01 versus the control group; ^#^ *p* < 0.05, ^##^ *p* < 0.01 versus the TBHP group.

**Figure S3.**Supplementation of βOHB alleviated OA progression in rats. (A) The schematic diagram of animal experiments. (B) The body weight of the rats was measured at various time points. (C) The βOHB levels in the synovial fluid were measured at different time points. (D) The serum βOHB levels were assessed at different time points. (E) The concentration of nitric oxide was quantified using Griess reagent. (F–H) The concentrations of IL‐6, TNF‐α, and PGE2 in the rat serum were determined through enzyme‐linked immuno sorbent assay. (I) The OARSI scores of knee cartilage were recorded. (J) Representative images of H & E staining were captured, with a scale bar of 100 μm. (K) Representative images of safranin O‐fast green staining were obtained, with a scale bar of 100 μm. *n* = 5. Data in (B–D) were analyzed by the two‐way ANOVA, data in (E–H) were analyzed by the one‐way ANOVA, and data in (I) were analyzed by the Kruskal–Wallis *H* test. *p < 0.05, **p < 0.01 versus the sham + SD group; ^#^ *p* < 0.05, ^##^ *p* < 0.01 versus the ACLT + SD group.

**Figure S4.**βOHB alleviated OA by activating mitophagy. The protein expression of HCAR2, IL6, Col2a1, MMP13, P16, P21, PINK1, Parkin, and P62 was detected by immunohistochemistry, scale bar 100 μm. *n* = 5. The obtained data was subjected to analysis using the one‐way ANOVA statistical method. *p < 0.05, **p < 0.01 versus the sham group; ^#^ *p* < 0.05, ^##^ *p* < 0.01 versus the ACLT group.

**Figure S5.**βOHB improves mitophagy through HCAR2. (A, B) Representative images of mitochondrial membrane potential assay were obtained using JC‐1, with a scale bar of 100 μm, *n* = 3. (C–F) Representative images and relative fluorescence intensity of PINK1 (red), LC3B (green), TOMM20 (yellow), and cell nuclei (blue) immunofluorescence were captured, with a scale bar of 50 μm, *n* = 5. (G) The ratio of tubular mitochondria and fragmented mitochondrial was observed based on the immunofluorescence staining results of TOMM20, *n* = 25. (H) Co‐localization of PINK1 (red), LC3B (green), TOMM20 (yellow). (I) Microstructural detection of mitophagy by transmission electron microscopy and normal mitochondria with high cristae abundance (green arrow), mitochondria with medium cristae abundance (yellow arrow), damaged mitochondria with low cristae abundance (red arrow), and autophagosomes with mitochondrial‐like organelles (blue arrow) were as indicated, scale bar = 10 μm. (J) The cristae abundance in mitochondria was quantified by the images of transmission electron microscopy, *n* = 25. (K) The ratio of cristae density and perimeter in mitochondria was quantified by the images of transmission electron microscopy, *n* = 25. (L, M) The percentage of damaged mitochondria and mitophagy events were quantified by the images of transmission electron microscopy, *n* = 25. The obtained data were subjected to analysis using the one‐way ANOVA statistical method. *p < 0.05, **p < 0.01 versus the siRNA‐scramble group; ^#^ *p* < 0.05, ^##^ *p* < 0.01 versus the TBHP + siRNA‐scramble group; ^&^ *p* < 0.05, ^&&^ *p* < 0.01 versus the TBHP + 4 mM βOHB + siRNA‐scramble group.

**Figure S6.**βOHB exerts chondroprotective effects via HCAR2 in TBHP‐induced chondrocytes. (A, B) The mRNA expression levels of P16, P21, IL6, MMP13, BAX, Col2a1, Aggrecan, BCL2, CAT, and SOD1 were evaluated by RT‐qPCR, *n* = 6. (C) The protein expression levels of P16, P21, iNOS, IL6, MMP13, cleaved PARP‐1, cleaved Caspase‐3, BAX, BCL2, CAT, SOD1, Pink1, Parkin, p‐Parkin, P62, and LC3B were evaluated by western blotting, *n* = 3. (D) Phosphorylated ubiquitin was detected by western blotting. (E) The relative protein expression levels of P16, P21, iNOS, IL6, MMP13, cleaved PARP‐1, cleaved Caspase‐3, BAX, BCL2, CAT, SOD1, Pink1, Parkin, p‐Parkin, P62, and LC3B were quantified by the ImageJ software (version 1.8). The obtained data were subjected to analysis using the one‐way ANOVA statistical method. *p < 0.05, **p < 0.01 versus the siRNA‐scramble group; ^#^ *p* < 0.05, ^##^ *p* < 0.01 versus the TBHP + siRNA‐scramble group; ^&^ *p* < 0.05, ^&&^ *p* < 0.01 versus the TBHP + 4 mM βOHB + siRNA‐scramble group.

**Figure S7.**Effects of βOHB on mitochondrial respiration and oxidative phosphorylation in chondrocytes. (A) Oxygen consumption rate (OCR) response in basal conditions and after consecutive addition of oligomycin 1.5 μM, FCCP 2.0 μM, and rotenone/antimycin A 0.5 μM, *n* = 3. (B–F) Quantification of mitochondrial basal respiration, maximal respiration, spare respiratory capacity, non‐mitochondrial oxygen consumption, and ATP production, *n* = 3. The obtained data were subjected to analysis using the one‐way ANOVA statistical method. *p < 0.05, **p < 0.01 versus the control group; ^#^ *p* < 0.05, ^##^ *p* < 0.01 versus the 15 μM TBHP group.

**Figure S8.** βOHB exerts protective effects on OA rats through the HCAR2/AMPK/PINK1 pathway. (A) Representative images of H & E staining were captured, with a scale bar of 100 μm. (B) Representative images of safranin O‐fast green staining were obtained, with a scale bar of 100 μm. (C) The OARSI scores of knee cartilage were recorded. *n* = 5. (D–I) The protein expression of HCAR2, p‐AMPK, PINK1, p‐Parkin, Parkin, and P62 was detected by immunohistochemistry, scale bar 100 μm. *n* = 5. The obtained data in (C) were analyzed by the Kruskal–Wallis *H* test. The obtained data in (D–I) were analyzed by the one‐way ANOVA. *p < 0.05, **p < 0.01 vs. the sham group; ^#^ *p* < 0.05, ^##^ *p* < 0.01 versus the ACLT group; ^&^ *p* < 0.05, ^&&^ *p* < 0.01 versus the ACLT + βOHB group.

## Data Availability

Data available on request from the authors.
