# Supplementary figures and images for: β‐Hydroxybutyrate enhances chondrocyte mitophagy and reduces cartilage degeneration in osteoarthritis via the HCAR2/AMPK/PINK1/Parkin pathway
Source: Aging Cell. 2024 Aug 9;23(11):e14294. doi: 10.1111/acel.14294 (PMC11561673; doi:10.1111/acel.14294)

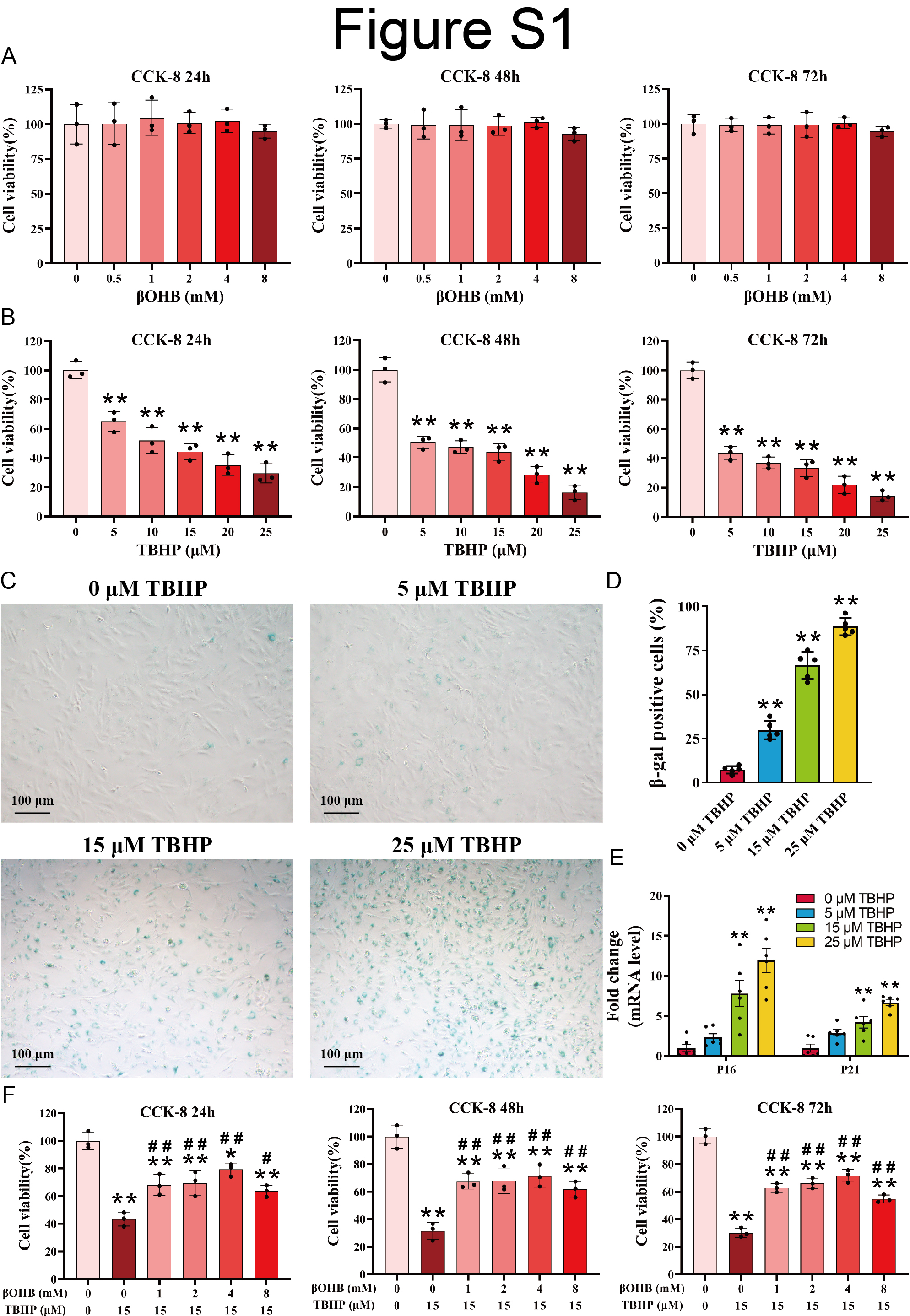

Supplement: Supplementary file 1 — Figure S1.Effect of βOHB on chondrocyte viability. (A, B) The cell counting kit‐8 assay was employed to measure the toxic effects of different concentrations of βOHB and TBHP on chondrocytes, n = 3. (C, D) SA‐β‐gal staining was utilized to observe the aging phenotype, with a scale bar of 100 μm, n = 3. (E) The mRNA expression of P16 and P21 was evaluated using RT‐qPCR, n = 6. (F) The toxic effects of 15 μM TBHP and varying concentrations of βOHB on chondrocytes were measured by cell counting kit‐8 assay, n = 3. The obtained data was subjected to analysis using the one‐way ANOVA statistical method. *p < 0.05, **p < 0.01 versus the control group; # p < 0.05, ## p < 0.01 versus the 15 μM TBHP group. [file ACEL-23-e14294-s006.jpg]

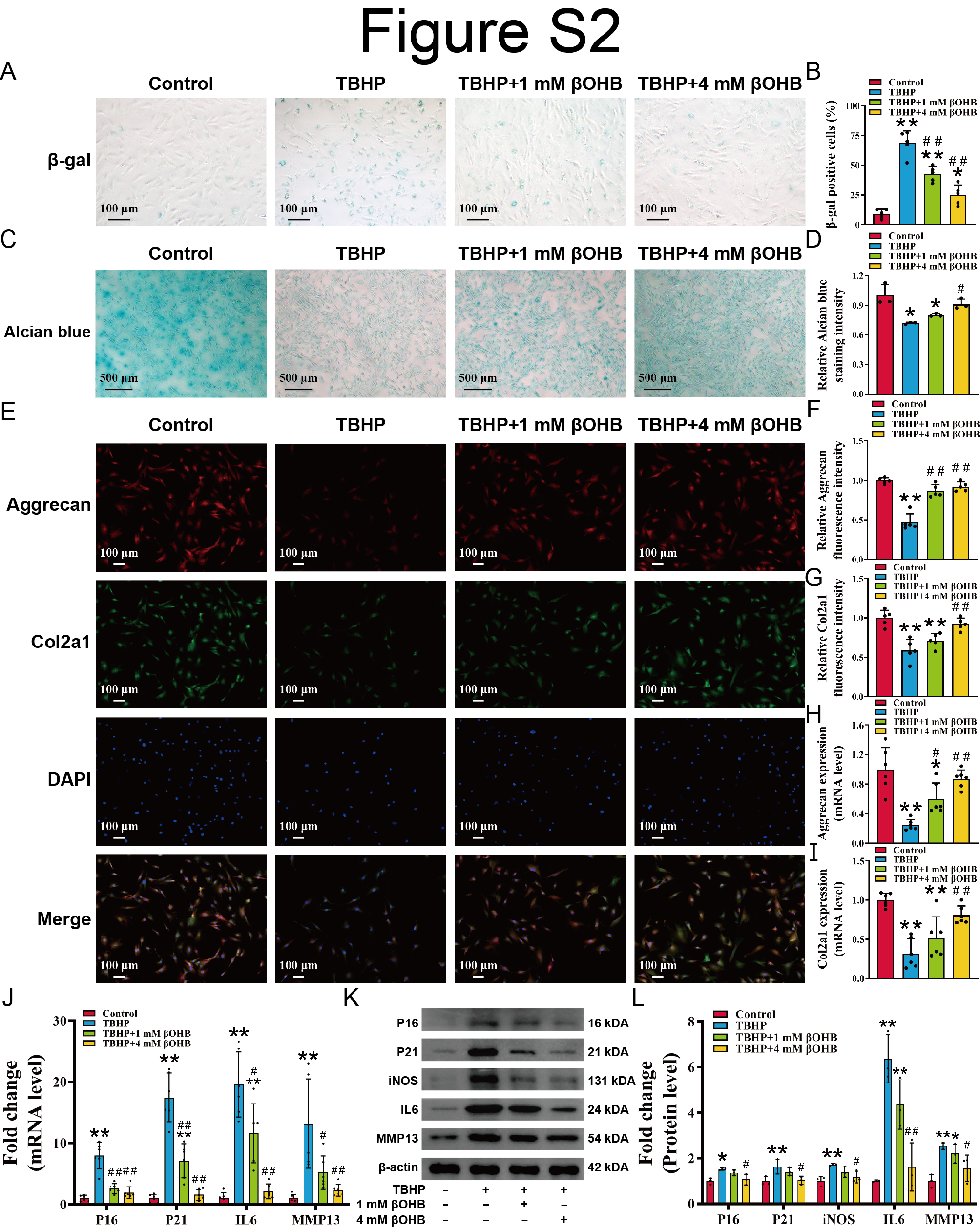

Supplement: Supplementary file 2 — Figure S2.βOHB attenuates TBHP‐induced senescence‐associated secretory phenotype in chondrocytes. (A, B) SA‐β‐gal staining was utilized to demonstrate the ratio of senescence chondrocytes, with a scale bar of 100 μm, n = 5. (C, D) Representative images of Alcian Blue staining were obtained, with a scale bar of 500 μm. (E–G) Representative images of Aggrecan (red), Col2a1 (green), and cell nuclei (blue) immunofluorescence were captured, with a scale bar of 100 μm, n = 5. (H–J) The mRNA expression of Aggrecan, Col2a1, P16, P21, IL6, and MMP13 was evaluated using RT‐qPCR, n = 6. (K, L) The protein expression of P16, P21, iNOS, IL6, and MMP13 was evaluated through western blotting, n = 3. The obtained data were subjected to analysis using the one‐way ANOVA statistical method. *p < 0.05, **p < 0.01 versus the control group; # p < 0.05, ## p < 0.01 versus the TBHP group. [file ACEL-23-e14294-s007.jpg]

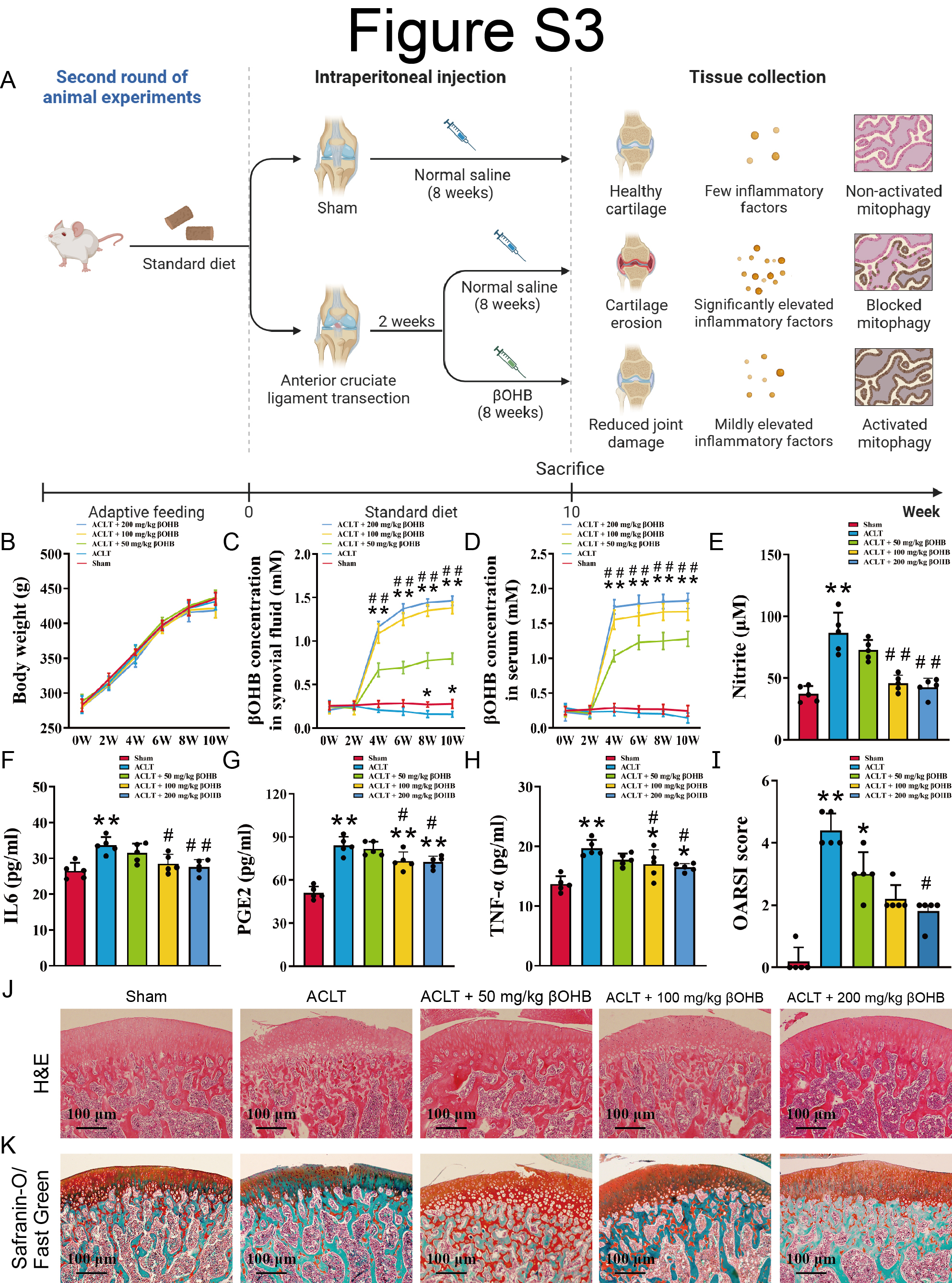

Supplement: Supplementary file 3 — Figure S3.Supplementation of βOHB alleviated OA progression in rats. (A) The schematic diagram of animal experiments. (B) The body weight of the rats was measured at various time points. (C) The βOHB levels in the synovial fluid were measured at different time points. (D) The serum βOHB levels were assessed at different time points. (E) The concentration of nitric oxide was quantified using Griess reagent. (F–H) The concentrations of IL‐6, TNF‐α, and PGE2 in the rat serum were determined through enzyme‐linked immuno sorbent assay. (I) The OARSI scores of knee cartilage were recorded. (J) Representative images of H & E staining were captured, with a scale bar of 100 μm. (K) Representative images of safranin O‐fast green staining were obtained, with a scale bar of 100 μm. n = 5. Data in (B–D) were analyzed by the two‐way ANOVA, data in (E–H) were analyzed by the one‐way ANOVA, and data in (I) were analyzed by the Kruskal–Wallis H test. *p < 0.05, **p < 0.01 versus the sham + SD group; # p < 0.05, ## p < 0.01 versus the ACLT + SD group. [file ACEL-23-e14294-s008.jpg]

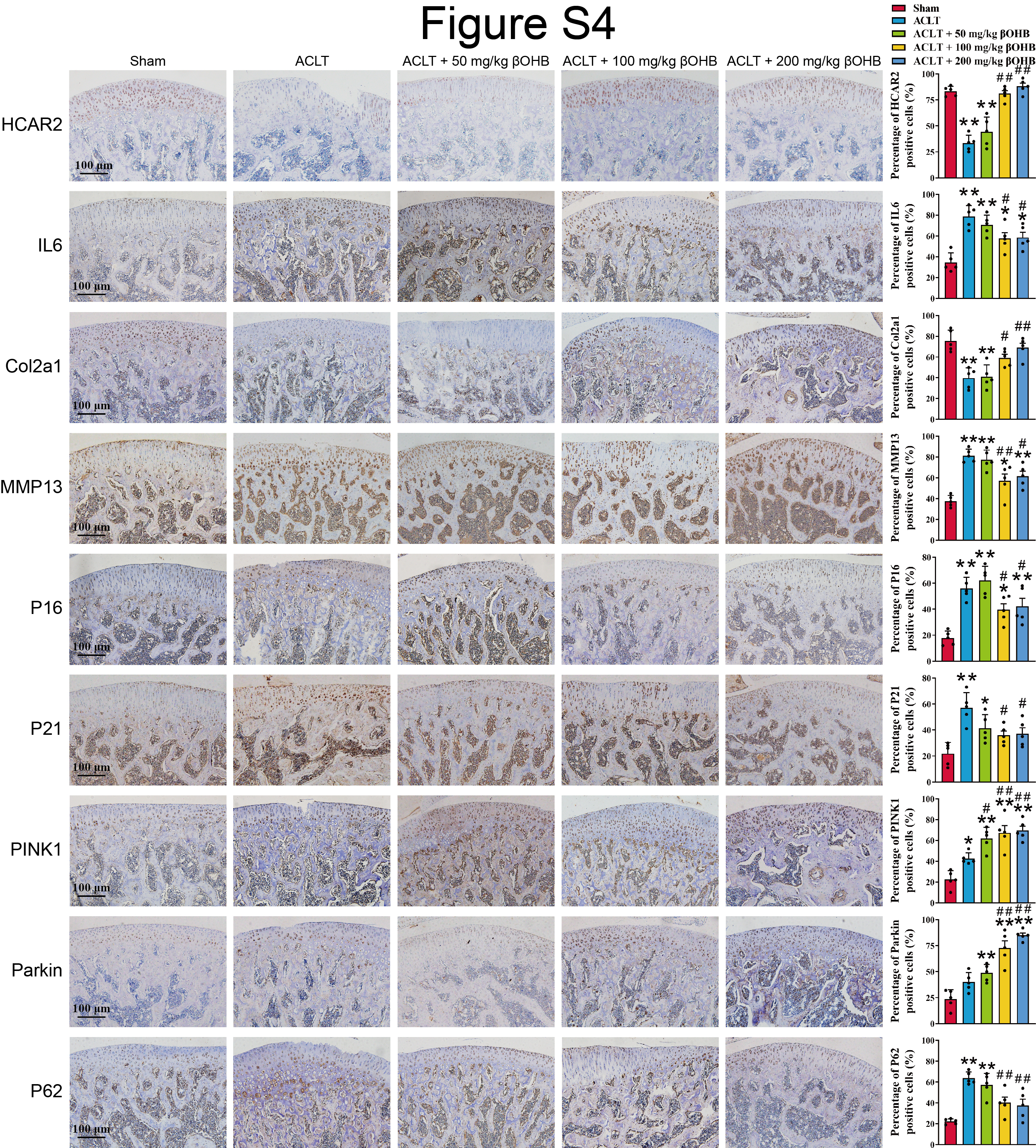

Supplement: Supplementary file 4 — Figure S4.βOHB alleviated OA by activating mitophagy. The protein expression of HCAR2, IL6, Col2a1, MMP13, P16, P21, PINK1, Parkin, and P62 was detected by immunohistochemistry, scale bar 100 μm. n = 5. The obtained data was subjected to analysis using the one‐way ANOVA statistical method. *p < 0.05, **p < 0.01 versus the sham group; # p < 0.05, ## p < 0.01 versus the ACLT group. [file ACEL-23-e14294-s005.jpg]

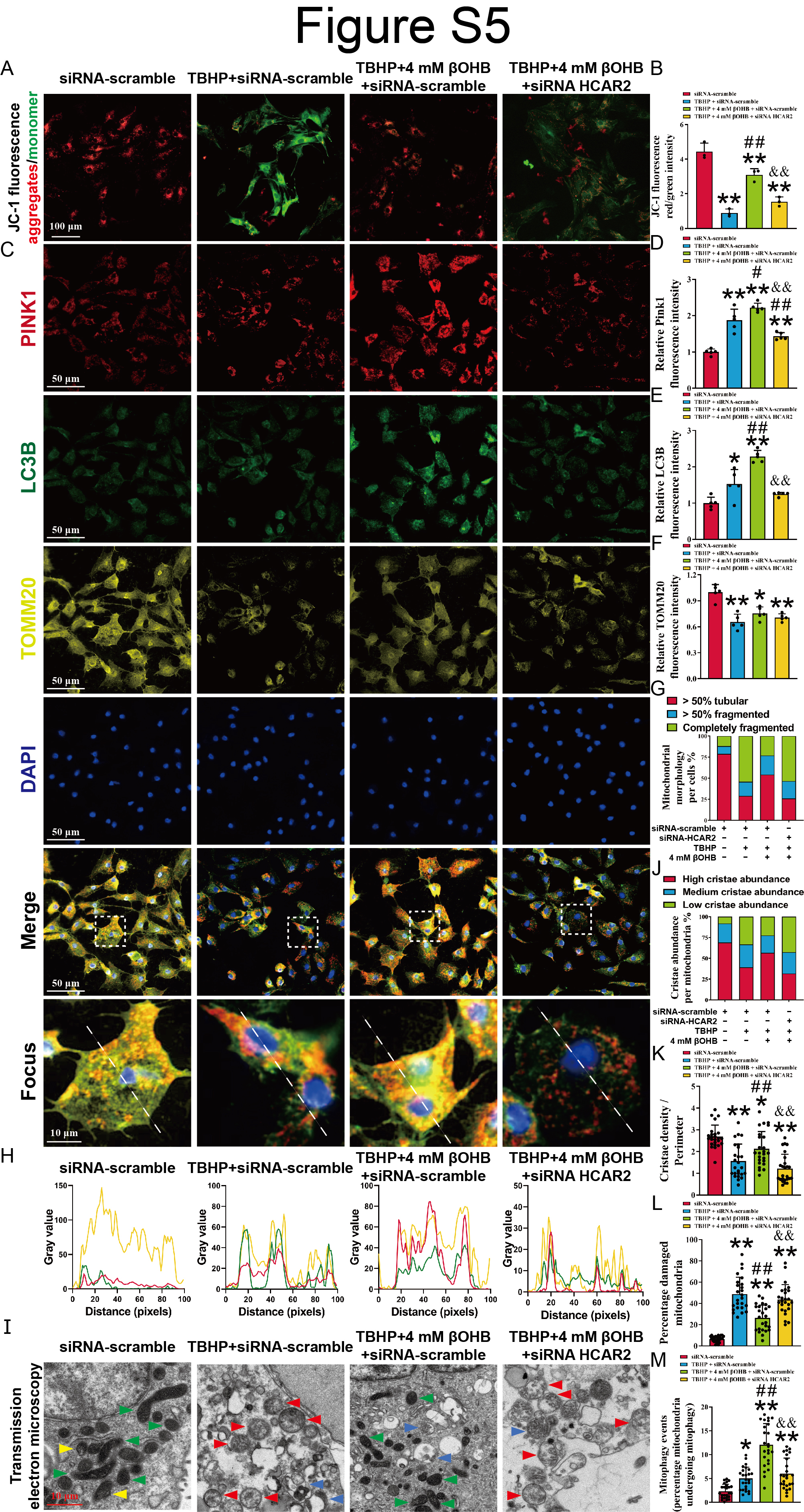

Supplement: Supplementary file 5 — Figure S5.βOHB improves mitophagy through HCAR2. (A, B) Representative images of mitochondrial membrane potential assay were obtained using JC‐1, with a scale bar of 100 μm, n = 3. (C–F) Representative images and relative fluorescence intensity of PINK1 (red), LC3B (green), TOMM20 (yellow), and cell nuclei (blue) immunofluorescence were captured, with a scale bar of 50 μm, n = 5. (G) The ratio of tubular mitochondria and fragmented mitochondrial was observed based on the immunofluorescence staining results of TOMM20, n = 25. (H) Co‐localization of PINK1 (red), LC3B (green), TOMM20 (yellow). (I) Microstructural detection of mitophagy by transmission electron microscopy and normal mitochondria with high cristae abundance (green arrow), mitochondria with medium cristae abundance (yellow arrow), damaged mitochondria with low cristae abundance (red arrow), and autophagosomes with mitochondrial‐like organelles (blue arrow) were as indicated, scale bar = 10 μm. (J) The cristae abundance in mitochondria was quantified by the images of transmission electron microscopy, n = 25. (K) The ratio of cristae density and perimeter in mitochondria was quantified by the images of transmission electron microscopy, n = 25. (L, M) The percentage of damaged mitochondria and mitophagy events were quantified by the images of transmission electron microscopy, n = 25. The obtained data were subjected to analysis using the one‐way ANOVA statistical method. *p < 0.05, **p < 0.01 versus the siRNA‐scramble group; # p < 0.05, ## p < 0.01 versus the TBHP + siRNA‐scramble group; & p < 0.05, && p < 0.01 versus the TBHP + 4 mM βOHB + siRNA‐scramble group. [file ACEL-23-e14294-s003.jpg]

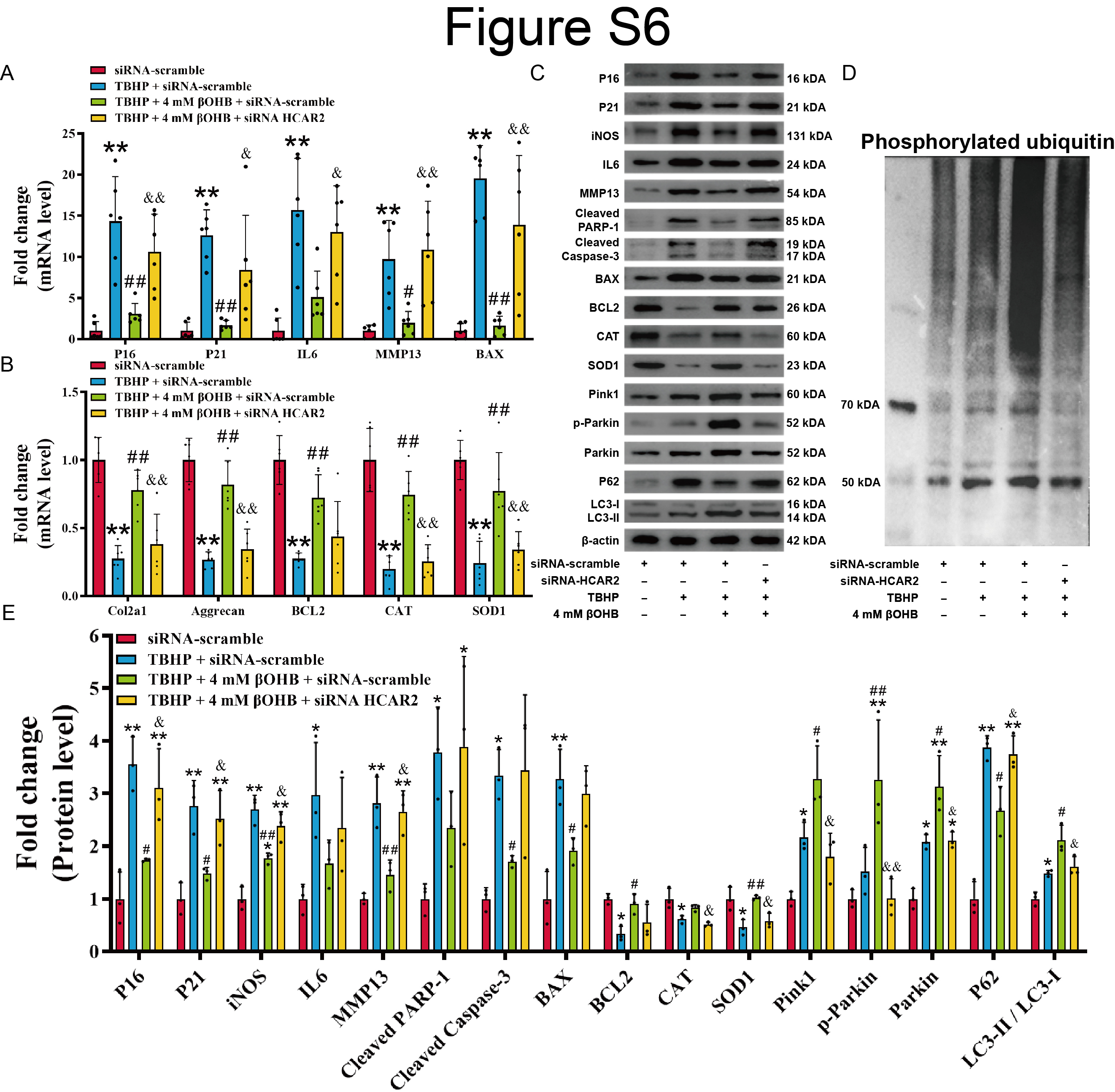

Supplement: Supplementary file 6 — Figure S6.βOHB exerts chondroprotective effects via HCAR2 in TBHP‐induced chondrocytes. (A, B) The mRNA expression levels of P16, P21, IL6, MMP13, BAX, Col2a1, Aggrecan, BCL2, CAT, and SOD1 were evaluated by RT‐qPCR, n = 6. (C) The protein expression levels of P16, P21, iNOS, IL6, MMP13, cleaved PARP‐1, cleaved Caspase‐3, BAX, BCL2, CAT, SOD1, Pink1, Parkin, p‐Parkin, P62, and LC3B were evaluated by western blotting, n = 3. (D) Phosphorylated ubiquitin was detected by western blotting. (E) The relative protein expression levels of P16, P21, iNOS, IL6, MMP13, cleaved PARP‐1, cleaved Caspase‐3, BAX, BCL2, CAT, SOD1, Pink1, Parkin, p‐Parkin, P62, and LC3B were quantified by the ImageJ software (version 1.8). The obtained data were subjected to analysis using the one‐way ANOVA statistical method. *p < 0.05, **p < 0.01 versus the siRNA‐scramble group; # p < 0.05, ## p < 0.01 versus the TBHP + siRNA‐scramble group; & p < 0.05, && p < 0.01 versus the TBHP + 4 mM βOHB + siRNA‐scramble group. [file ACEL-23-e14294-s001.jpg]

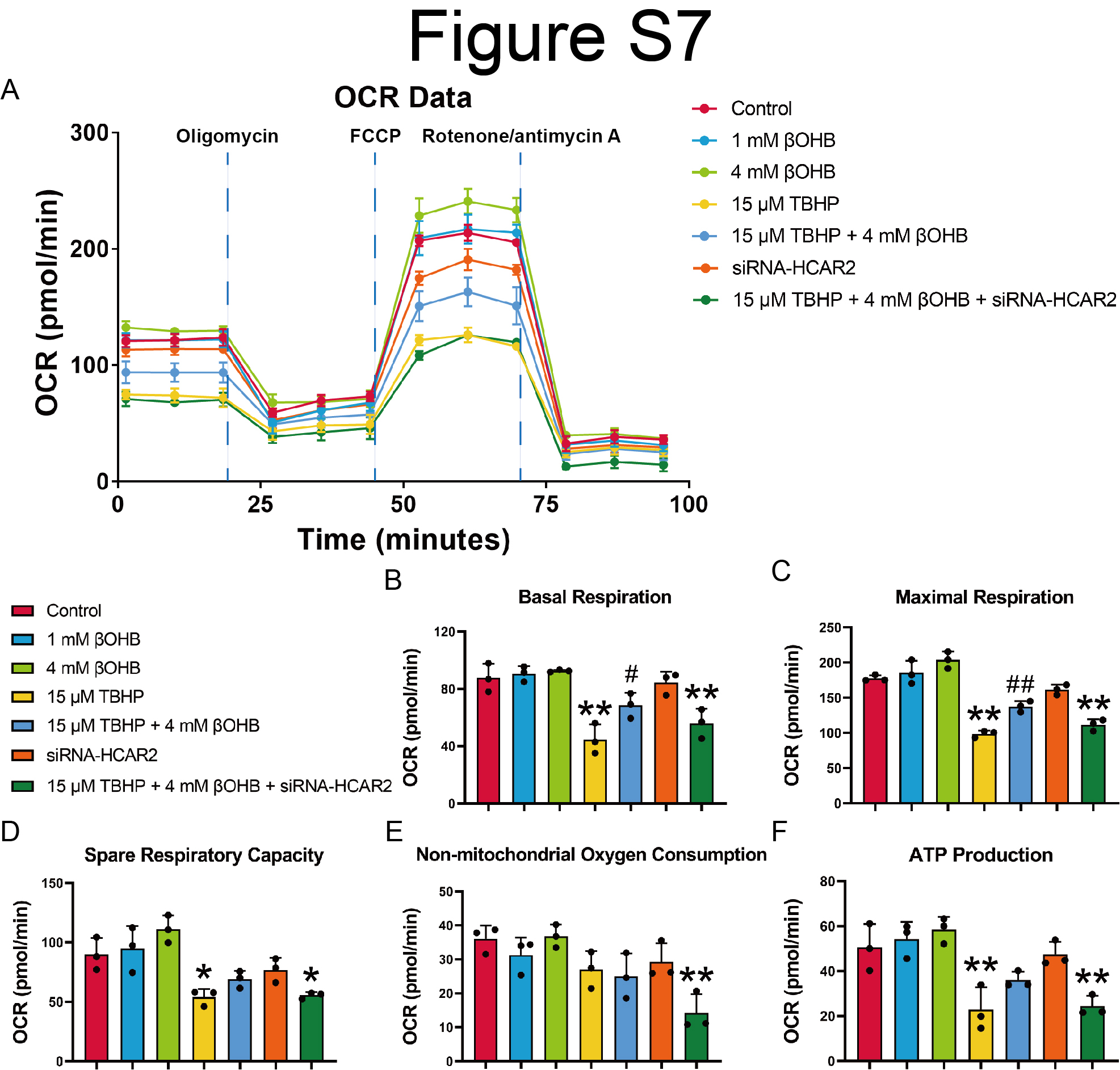

Supplement: Supplementary file 7 — Figure S7.Effects of βOHB on mitochondrial respiration and oxidative phosphorylation in chondrocytes. (A) Oxygen consumption rate (OCR) response in basal conditions and after consecutive addition of oligomycin 1.5 μM, FCCP 2.0 μM, and rotenone/antimycin A 0.5 μM, n = 3. (B–F) Quantification of mitochondrial basal respiration, maximal respiration, spare respiratory capacity, non‐mitochondrial oxygen consumption, and ATP production, n = 3. The obtained data were subjected to analysis using the one‐way ANOVA statistical method. *p < 0.05, **p < 0.01 versus the control group; # p < 0.05, ## p < 0.01 versus the 15 μM TBHP group. [file ACEL-23-e14294-s002.jpg]

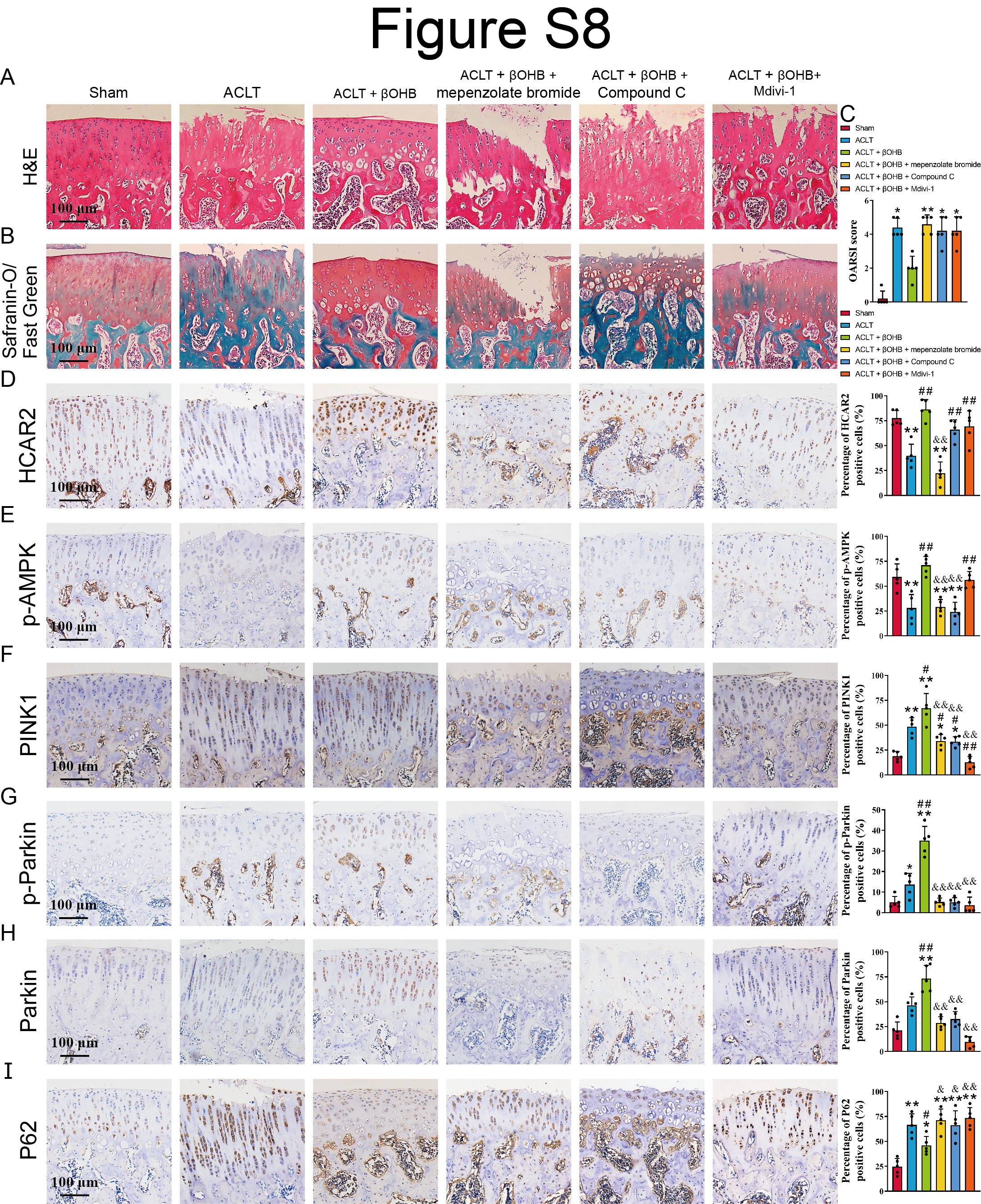

Supplement: Supplementary file 8 — Figure S8. βOHB exerts protective effects on OA rats through the HCAR2/AMPK/PINK1 pathway. (A) Representative images of H & E staining were captured, with a scale bar of 100 μm. (B) Representative images of safranin O‐fast green staining were obtained, with a scale bar of 100 μm. (C) The OARSI scores of knee cartilage were recorded. n = 5. (D–I) The protein expression of HCAR2, p‐AMPK, PINK1, p‐Parkin, Parkin, and P62 was detected by immunohistochemistry, scale bar 100 μm. n = 5. The obtained data in (C) were analyzed by the Kruskal–Wallis H test. The obtained data in (D–I) were analyzed by the one‐way ANOVA. *p < 0.05, **p < 0.01 vs. the sham group; # p < 0.05, ## p < 0.01 versus the ACLT group; & p < 0.05, && p < 0.01 versus the ACLT + βOHB group. [file ACEL-23-e14294-s004.jpg]
